# Joint zonated quantification of multiple parameters in hepatic lobules

**DOI:** 10.1038/s41598-026-46721-5

**Published:** 2026-05-14

**Authors:** Hendrik Laue, Daniel Budelmann, Mohamed Albadry, Christiane Engel, Nick Weiss, Uta Dahmen, Lars Ole Schwen

**Affiliations:** 1 Fraunhofer MEVIS, Max-von-Laue-Str. 2, 28359 Bremen, Germany; 2 Fraunhofer MEVIS Lübeck, Maria-Goeppert-Straße 3, 23562 Lübeck, Germany; 3 Experimental Transplantation Surgery, Department of General, Visceral and Vascular Surgery, University Hospital Jena, Jena, Germany; 4 Department of Pathology, Faculty of Veterinary Medicine, Menoufia University, Shebin Elkom, Menoufia Egypt

**Keywords:** Image analysis, Zonated quantification, Histological images, Liver, Steatosis, Cytochrome P450, Clinical microbiology, Diagnostic markers, Preclinical research, Experimental models of disease, Alcoholic liver disease, Non-alcoholic fatty liver disease, Hepatocytes

## Abstract

Many liver diseases have a distinct zonation pattern. Similarly, most metabolic processes in the hepatic lobule are also spatially organized. Understanding the interplay between a zonated disease pattern and its impact on zonated metabolic liver function requires the joint quantification of both phenomena. Our study presents an image analysis workflow for the joint zonated quantification of multiple parameters from whole-slide images of conventionally stained serial sections of mouse livers. As a proof of concept, we used small stacks of six adjacent sections, differentially stained with HE, GS and four different CYP enzymes from three mice with different severities of steatosis. Portal fields and central veins were annotated and transferred to adjacent slide images via image registration. The result was visually confirmed to avoid errors in the non-rigid transformation process. This approach allowed identifying the same lobules and zones in multiple consecutive sections. Zones were obtained by arbitrarily dividing the distance between portal fields and central veins into twelve intralobular zones. Zonal distributions of various parameter combinations quantified from multiple slides were visualized in the geometry of lobules and zones and plotted as scatter diagrams. Using this workflow, we could visualize differences in the heterogeneous expression patterns of marker proteins in normal and steatotic livers. As a next step, the algorithm presented here can be applied to a scientific question such as quantifying the impact of zonated steatosis of different severity on the zonated expression pattern and the resulting metabolic function of CYP enzymes.

**Supplementary Information:**

The online version contains supplementary material available at 10.1038/s41598-026-46721-5.

## Introduction

**Fig. 1:**
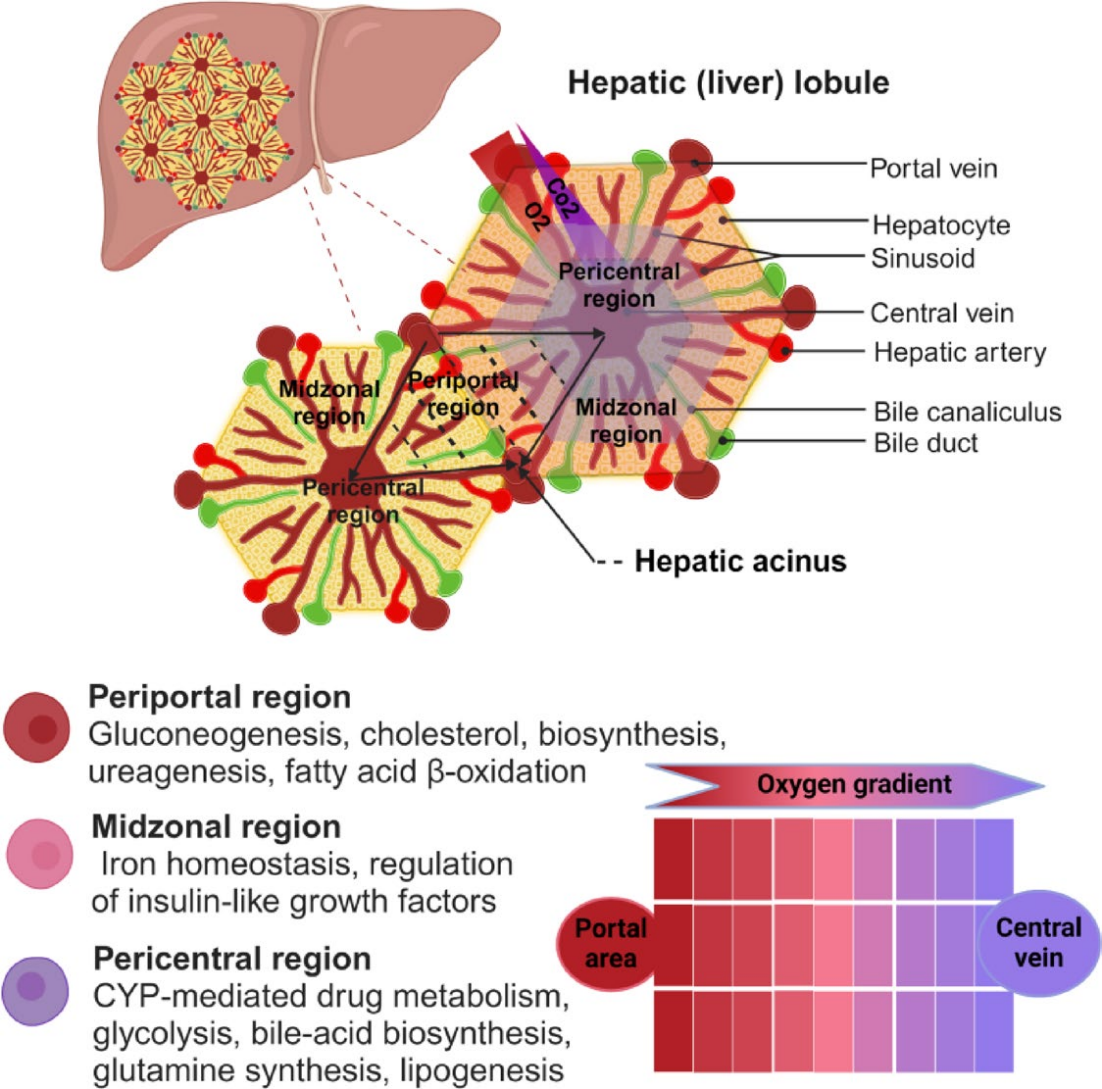
Hepatic lobular structure and zonation. ‘‘Created with BioRender.com”. Adapted from Albadry 2024^10^.

The liver is organized in lobules^1,2^ that consist of hepatic cords originating at the portal triad on the periphery of the lobule to the central vein in the center of the lobule. Hepatocytes are arranged in alignment with the afferent vascular systems, including the portal veins and hepatic arteries, as they merge into the hepatic capillaries, known as hepatic sinusoids (see Fig. 1)^3,4^.

The hepatic lobule exerts many metabolic processes that are spatially arranged, a phenomenon known as metabolic zonation^5^. Metabolic zonation in hepatic lobules was first described by Gebhardt 1993^5^ and later by Lindros 1997^6^. The lobule is typically divided into three regions called periportal, midzonal, and pericentral zone (see Fig. 1).

Metabolic zonation occurs because of the intralobular gradient of oxygen and nutrients along the porto-central axis. The hepatocytes close to the portal field are exposed to blood rich in oxygen and nutrients, performing rather anabolic functions such as gluconeogenesis and protein synthesis. In contrast, the hepatocytes close to the central vein are exposed to less oxygen and nutrients and are more specialized in catabolic processes such as glycolysis and xenobiotic metabolism^7,8^.

Metabolic zonation can be assessed by visualizing the spatial distribution of zonated biomarkers such as E-cadherin as a periportal marker^7^ and Glutamine synthetase (GS) and Cytochrome P450 (CYP) enzymes as pericentral proteins^9^. Zonated quantification calls for accurately determining spatially distributed positive signals along the porto-central axis.

Zonated quantification of biomarkers is an essential aspect of liver disease research, as many diseases manifest in a non-uniform manner throughout the hepatic lobule.

Non-alcoholic fatty liver disease (NAFLD) is one of the disorders belonging to the spectrum of liver diseases which frequently progresses in a zonated manner^11^. Non-alcoholic steatohepatitis (NASH)/cirrhosis and NAFLD begin with hepatic steatosis and inflammation in the pericentral region and spread outward, respectively^12^^[,13^. Other liver diseases are rather confined to the periportal areas, such as autoimmune hepatitis^14^, primary biliary cirrhosis^15^, and iron overload-induced injury^16^.

To better understand the interplay between zonated liver disease and zonated expression of biomarkers, visualization and zonated quantification of both are needed. Joint zonated quantification can be achieved by multiplex fluorescent staining of multiple biomarkers in the same section. However, fluorescent staining is inferior regarding the morphological assessment of altered tissue structures. Here, conventional brightfield microscopy is more suitable. Furthermore, scanning devices acquiring multiplex fluorescent staining are still rare due to the enormous high cost of investment.

Therefore, it is essential to develop an image analysis approach that can jointly quantify the spatial distribution of zonated disorders and the zonated expression of biomarkers in WSI from adjacent sections subjected to Hematoxylin-eosin (H&E) and conventional immunohistochemistry (IHC) staining. Up to now, different image analysis approaches have been developed for zonated quantification of morphological changes and for differentially expressed biomarkers, visualized by immunohistochemical staining.

Schwen et al. developed a semi-automatic image analysis approach to quantify zonation and spatial distribution of steatosis in a murine liver^17^. Steatosis in lobules was quantified using relative distances between vessel positions^17^. For this purpose, the interval between portal fields (PF) and central veins (CV) was divided arbitrarily into twelve zones spanning over the three regions of the hepatic lobule, allowing a refined evaluation of the spatial distribution of steatosis. Clustering of the lobule-wise zonation patterns was used to identify typical and substantially different patterns.

Peleman and co-workers quantified the zonated distribution of the hypoxia marker pimonidazole in the normal and steatotic liver^18^. They employed Voronoi distance from the central vein to estimate the area of the lobules. The location of CV, as one key vessel determining the hepatic lobule, was marked on the IHC image using GS, a biomarker with stable expression in the pericentral region. In contrast, the periportal region was manually annotated using images from H&E-stained sections.

Rong et al. implemented a deep learning approach called “Tissue Positioning System” to automatically determine the spatial distribution of different marker proteins within the mouse liver lobule^19^.

Recently, we developed an image analysis pipeline using ​​the Virtual Alignment of Pathology Image Series (VALIS)^9,20^ method for image registration and generation of multiplexed protein WSI. This approach was applied to automatically quantify the normalized intensity of various marker proteins and geometrical parameters in the normal livers of four distinct species (mice, rats, pigs and humans)^9^.

This multi-parameter investigation conducted by Albadry et al. 2024^9^ used neighboring sections, differentially stained with H&E, GS, as well as the key CYP450 enzymes. Data were registered in a superpixel format, resampling the images with a common pixel geometry. The lobules, zones and vessels were detected by the super-pixel segmentation from OpenCV followed by KMeans clustering. The lobule borders were generated from the combined intensities of all acquired images. Zonation was defined by the distance to the pericentral zone. For the detection of CYP, color deconvolution^21^ was used.

A local comparison of multiple histological parameters in tumors by destaining and re-staining sections was conducted by Remark et al. 2016^22^. Similarly, Lotz et al. 2023^23^ compared consecutive and re-stained sections for image registration in histopathology, as registering the images is still necessary for re-stained sections.

In summary, different methods for zonated quantification were established. However, joint quantification of zonated morphological disorders and zonated expression of biomarkers still needs to be done. This knowledge is essential for better understanding the influence of zonated morphological disorders like steatosis and fibrosis on metabolic zonation.

Here, we describe a method for joint quantification of morphological changes and biomarkers from adjacent, differently stained serial histological sections derived from the same tissue sample.

To showcase this technique, we jointly quantified periportal steatosis and several pericentrally expressed drug metabolizing enzymes (CYP) and GS in carefully selected consecutive sections from one normal liver and two mouse livers with different severity of steatosis.

Our approach will eventually allow better understanding and visualization of the interplay of different zonated hepatic pathologies and marker proteins while also assessing heterogeneity at the liver lobule and liver lobe scales.

## Methods

### Histological images

We used selected stacks of images from the previously mentioned experiment to showcase the image analysis pipeline^24^. In this experiment, whole slide scans were generated from six differently stained adjacent histological sections from normal and steatotic male mouse livers (*n* = 6). Hepatic steatosis was induced by feeding the mice for either 2 or 4 weeks a methionine-choline deficient but high-fat diet, as described in Albadry 2022^24^. At the end of the respective feeding period, the animals were euthanized under 5% isoflurane anesthesia and pre-emptive analgesia using buprenorphine in a dose of 0.05 mg/kg body weight applied 30 min prior to laparotomy, as approved by the local authorities (UKJ-19–020). After opening the abdomen, the liver was explanted and four liver lobes (left lateral lobe, right median lobe, right superior lobe, caudate inferior) were fixed in 5% formaldehyde and parafin-embeded. Consecutive histological sections of 3 μm thickness were cut for H&E and IHC staining using antibodies for GS and four CYP enzymes (CYP1A2, CYP2D6, CYP2E1, and CYP3A4) as described before by Albadry et al. 2022^24^. All slides were scanned at 200x magnification using a Hamamatsu NanoZoomer HT 2.0 slide scanner (L11600, Hamamatsu City, Japan), resulting in a resolution of 0.227 μm/px. Imaging data is available from Albadry et al. 2022^24^.

We used one exemplary sequence of consecutive whole slide scans representing the four liver lobes from one animal from each of the three experimental groups (control, 2 weeks and 4 weeks feeding the diet). The small stacks were selected based on section and image quality (no or little folds or tears in the section, homogenous staining quality) and successful registration of the six consecutive sections subjected to differential staining. Depending on the size of the tissue, we analysed between 175 and 295 lobules per mouse liver section.

### Ethics statement

The animal experiment and housing were conducted in compliance with the prevailing German rules and guidelines for animal care and the ARRIVE guidelines for reporting animal research. The animal study was approved by the Thüringer Landesamt für Verbraucherschutz, Thuringia, Germany, with the approval number UKJ-19–020.

### Registration of whole-slide images

**Fig. 2:**
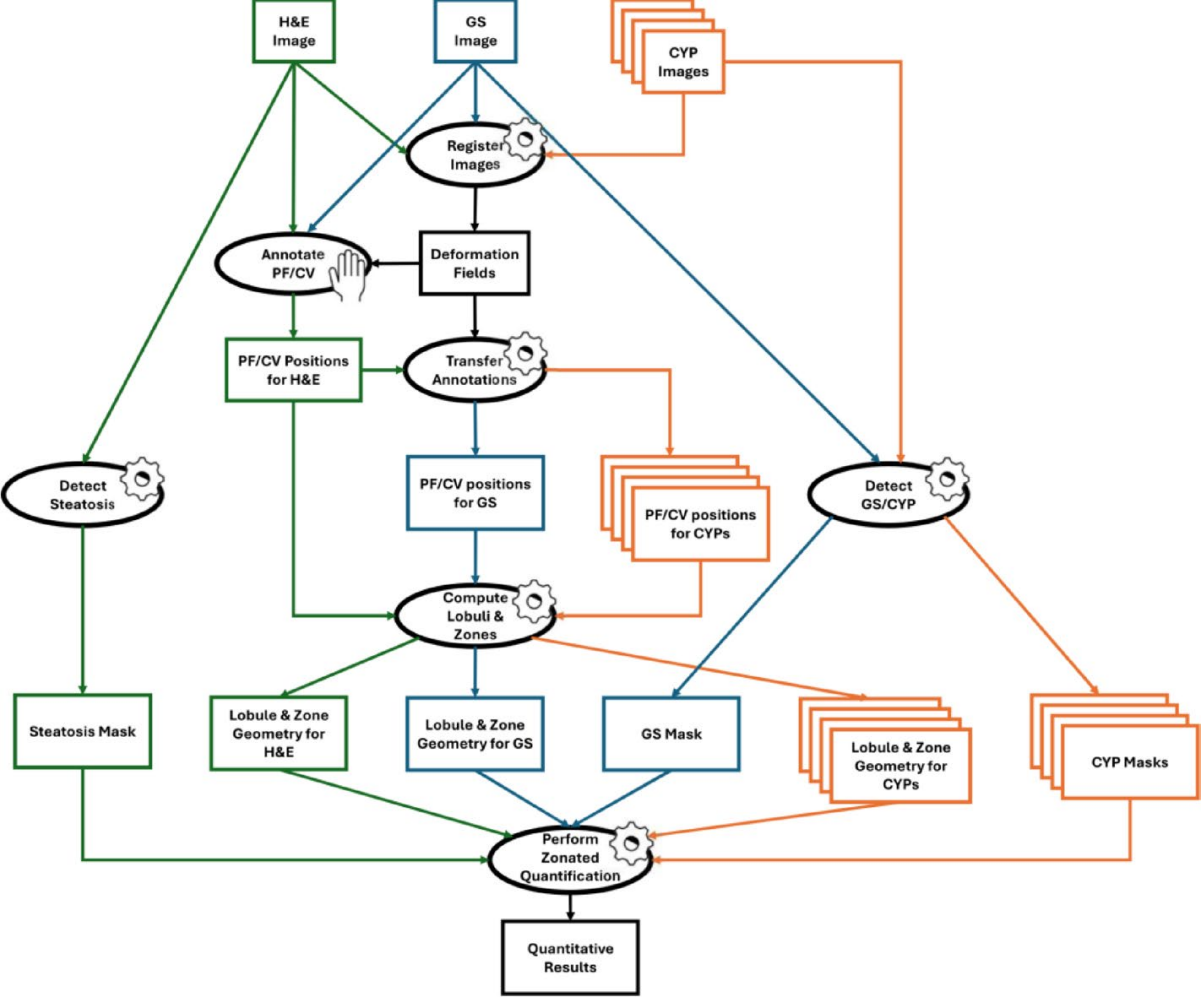
Flow chart showing the process of the Zonated Quantification. Boxes indicate data, ellipses represent processes (cogwheel: fully automatic, hand: with manual steps). The images are registered, the resulting deformation field is used to display the aligned GS and H&E. Portal fields (PF) and central veins (CV) are annotated on the aligned display. The annotations are transferred to each image in the stack in order to compute the lobules and zones. Steatosis and GS/CYP signals are detected directly in the images. The detected signals are then quantified in the computed lobules and zones, yielding quantitative results as a table. This data can subsequently be plotted, used for descriptive analysis, evaluated in downstream analyses, or used to parameterize computational models.

The images were registered using pre-alignment, rigid, and non-rigid registration according to Lotz et al. 2023^23^. The registration order followed the staining sequence of the mini stack starting with GS, followed by CYP1A2, H&E, CYP2D6, CYP3A4, and CYP2E1. Hence, the GS WSI was first used as a reference (fixed) image, and CYP1A2 as the template (moving) image for non-rigid registration. Then, the deformed CYP1A2 image was a reference to the H&E template, the deformed H&E was a reference to the CYP2D6 template, etc. Using directly adjacent pairs of reference and template images for the image registration minimized morphological differences between the slides, which enhanced the registration result, see Lotz et al. 2014^25^. This resulted in coordinate transformation fields H&E → GS and CYP* → GS for each CYP, mapping each slide into the same coordination system with GS remaining untransformed. We applied these transformations for two purposes: to view the H&E slide aligned to the GS slide for annotating PF and CV, and to transform annotations to each slide for computing lobules and zones. The entire image analysis pipeline is illustrated in Fig. 2.

### Annotation of portal and central veins

The portal field and central veins were annotated on the deformed H&E slides, using axis-aligned rectangles as described in Budelmann et al.^26^. In this process, distinguishing PFs and CVs was facilitated by toggling between viewing H&E and GS images, which were displayed aligned using the coordinate transformation H&E → GS. The PF and CV annotations were transformed automatically to the CYP images by applying the inverted transformations GS → CYP* to each rectangle vertex.

The subsequent lobule computation is based on the approximate center points of PF and CV. Thus, we computed the center points of each annotated polygon. To obtain a 1:1 correspondence of lobules, we kept track of the transformed annotations via unique identifiers (pseudo-random v4 UUIDs^27^.

### Detection of steatosis and marker proteins

Starting from the original resolution of 0.227 μm per pixel, the following steps used a coarser resolution of the WSI. To keep the computing time and memory requirements within reasonable limits (< overnight for computing, < 64GB memory), the images were analyzed at a quarter of the original resolution, i.e., at 0.909 μm per pixel.

For macrovesicular steatosis detection, we obtained a binary mask representing macrovesicular fat droplets using the fat detection algorithm described in Homeyer et al. 2015^28^.

The GS and CYP quantification comprise tissue mask generation, classification, and quantification. A rough binary tissue mask was generated using Otsu’s methods on the grayscale WSI, as described in Budelmann et al., 2022 and Otsu et al., 1979^26,29^. Then, we divided the 3D (RGB-) histogram of the tissue-masked image into three classes using expectation maximization^30^. The mean color values of the classes were used to classify the nearest neighbouring class to each color value in the image as a tissue type. This way, the image areas corresponding to the classes approximate positively and negatively stained tissue and unstained areas (i.e., lipid vacuoles, blood vessels, and tissue outside the respective liver lobule). A ternary mask was generated by classifying each pixel into one of the three classes: ‘unstained’, ‘positive’, and ‘negative’.

### Determination of zones and lobules

Lobule and zone masks were computed from the transformed PF and CV coordinates for each HE, GS, and CYP image separately, using the methods described in Schwen et al. 2016^17^: Lobules were identified by using the portality for the image area, defined by the distance to the closest PF and CV coordinates as

1 $$\:portality\left(x\right):=1 - \frac{dist(x,\:closest\:PF)}{dist(x,\:closest\:PF)\:+\:dist(x,\:closest\:CV)}$$

for every point *x*. Lobules were computed as the catchment basins of a pre-flooded watershed transform^31^ of the portality, and each lobule was assigned an ascending index. The zones were then defined by quantizing the portality into twelve intervals, thus dividing each lobule into twelve zones and assigning indices one to twelve.

The lobules and zones were stored in two maps with labels for the zone index and each lobule index. These maps were generated for the H&E-, GS-, and CYP-stained sections, which provided zone and lobule information for each pixel in these images.

### Zonated quantification

The steatosis was quantified as the fat droplet area fraction computed for each zone in each lobule. The quantification of CYP and GS expression was done similarly with the exception of a minimum threshold of 5% ‘stained’ tissue. This allows suppressing noisy data in void and unstained areas. In the remaining zones, the ratio of ‘positively stained tissue’ to ‘all tissue’ (positively + negatively stained tissue) was computed based on the ternary mask determined above. These analyses were implemented using Python and OpenCV^32^.

### Visualization

Results of zonated quantification of single and jointly quantified parameters were visualized and shown qualitatively in two-channel color maps representing the geometric lobular location, with blue representing steatosis, red the target protein, and thus magenta the joint presence of both signals and black the absence of specific signals.

The distribution of measurements determined from the zonated quantification were shown using box-and-whisker plots for single parameters and scatter plots for pairs of jointly quantified parameters. These give a quantitative overview over the variability within the same zone across all lobules (width of the boxes/area covered by color-coded groups of points in the scatter plots); and differences between zones combining the information of all lobules (differences between boxes/areas covered by different groups of points).

### Trend characterization

We employed a linear regression model to characterize the spatial distribution of the CYP enzymes and steatosis across the hepatic lobule in three different mice with different feeding periods.

For this purpose, we modeled the presence of steatosis or enzyme p as a linear function of the lobular position *x*, such that $p(x){\text{ }} = {\text{ }}m \cdot x + p_{0}$, where *m* represents the slope and *p*_*0*_ the p-axis intercept. The lobular position *x* was normalized from 0 (portal field) to 1 (central vein), enabling a standardized comparison of spatial gradients independent of the number of zones.

Model fitting was performed individually for each lobule and each measured parameter by minimizing the squared error between observed and predicted values, using NumPy’s polyfit function (version 1.25.1). We calculated the mean and standard deviation of the resulting slopes and intercepts of all lobules for each mouse. These values were visualized using box-whisker plots.

Finally, we quantify the observed relationship between steatosis and CYP/GS expression across all lobules for the different mice by employing a similar approach: We expressed the CYP/GS presence *p* as a function of steatosis *s*, following the equation $p(s) = m_{s} \cdot s + p_{{s,0}}$, where *m*_*s*_ is the slope and *p*_*S,0*_ is the *p*_*S,0*_-axis intercept representing CYP/GS presence levels for absence of steatosis. The resulting slopes and intercepts for the combinations were visualized using box-whisker plots.

## Results

Using zonated quantification in the hepatic lobule allowed visualizing the relation between the spatial distribution of steatosis and the expression of marker proteins, specifically drug-metabolizing CYP enzymes. We included GS staining in our analysis as a plausibility check.

The results of the joint assessment of steatosis and biomarker expression are presented in two different ways in addition to the native tabular format. The visualization using combined color maps projects the results on the lobular tissue geometry. Median, quartiles, minimum and maximum values as well as outliers of the zonated quantification of single- and dual parameters for all lobules are shown in box-and-whisker plots and scatter diagrams.

### Joint quantification of multiple parameters

Steatosis, GS and CYP expression for all zones and all lobules from one WSI representing a steatotic mouse liver section are shown in Table 1.

**Table 1:** Results of joint zonated quantification for six adjacent WSI. For each lobule and each zone, multiple parameters were quantified from adjacent serial sections: steatosis as the area fraction detected as macrovesicular lipid vacuoles of the zone; GS and CYP expression as the ratio of positively stained over positively and negatively stained tissue. Due to the impact of vessel area, zone 1 and 12 have been omitted. The full table is available in the Zenodo repository.

Lobule	Zone	Steatosis	GS	CYP1A2	CYP2D6	CYP2E1	CYP3A4
51a…75	2	0.0231	0.4069	0.7272	0.7422	0.8135	0.8684
	3	0.0512	0.2987	0.6450	0.7551	0.8256	0.8632
	⋮	⋮	⋮	⋮	⋮	⋮	⋮
	11	0.2028	0.0005	0.0723	0.5893	0.1086	0.6710
832…5d	2	0.0013	0.4679	0.7415	0.7748	0.7926	0.8495
	3	0.0212	0.2632	0.6011	0.7539	0.7562	0.8331
	⋮	⋮	⋮	⋮	⋮	⋮	⋮
	11	0.1702	0.0194	0.0448	0.6706	0.1073	0.6709

The parameters from each staining were matched to the associated lobule and zone via the ID of the corresponding central vein. These visualizations are displayed at a resolution of 3.7 μm per pixel, i.e., the image analysis results were downsampled by a factor of four in both dimensions using standard nearest-neighbor interpolation.

**Fig. 3:**
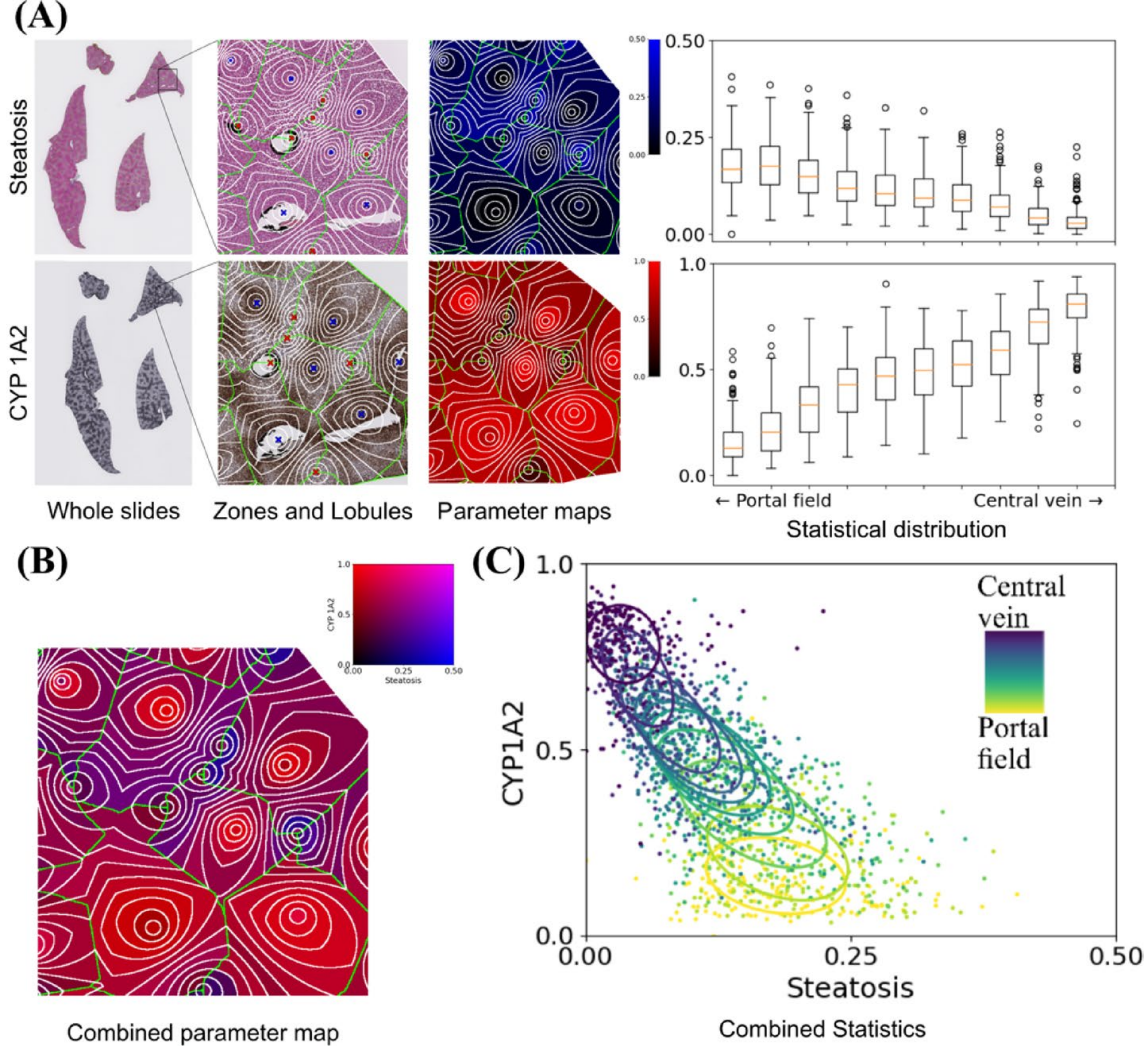
Overview of the heterogeneous dual parameter representation for all four liver lobes evaluated using the combined steatosis and CYP1A2 parameter map. The variable dual color intensity with areas in red and blue with others showing magenta indicates a heterogeneous signal pattern within the section. The missing lobules in the two liver lobes shown on the right side are caused by central veins without portal fields in their vicinity. These were probably missed due to the three-dimensional structure of the lobules and possible tissue distortions caused by cutting.

The steatosis presence in the zones was displayed by the blue intensity in the RGB color model and the CYP/GS presence by the red intensity, respectively (see Fig. 3A).

In the next step, we created the visualization using dual-channel color maps where the morphological changes in steatosis were depicted in blue and the CYP/GS enzymes in red. Using the CYP2A1 staining of a steatotic mouse liver as an example shows heterogeneous appearance of the four false marker colors, suggesting a heterogeneous presence of steatosis and CYP expression, respectively (see Fig. 3B).

Joint visualization of the quantified signals was achieved by creating a combined scatter plot depicting the parameter obtained from morphology on the horizontal axis and one parameter obtained via IHC staining on the vertical axis. For each data point showing the severity of steatosis and the extent of staining, the corresponding zone is represented by a viridis color map with dark blue representing the pericentral zones and yellow representing the periportal zones. To characterize the distribution and show differences between zones, one ellipse per zone indicates the mean value and 2D standard deviation of the values for all lobules.

Using CYP1A2 expression in a steatotic mouse liver serves as a showcase to visualize the relationship between the periportally located steatosis and the intense expression of CYP1A2 in the pericentral region.

This zonal distribution is illustrated by the dark blue ellipses (pericentral zone) in the upper left quadrant and the yellow ellipses (periportal zone) in the lower right corner of the diagram (see Fig. 3C).

**Fig. 4:**
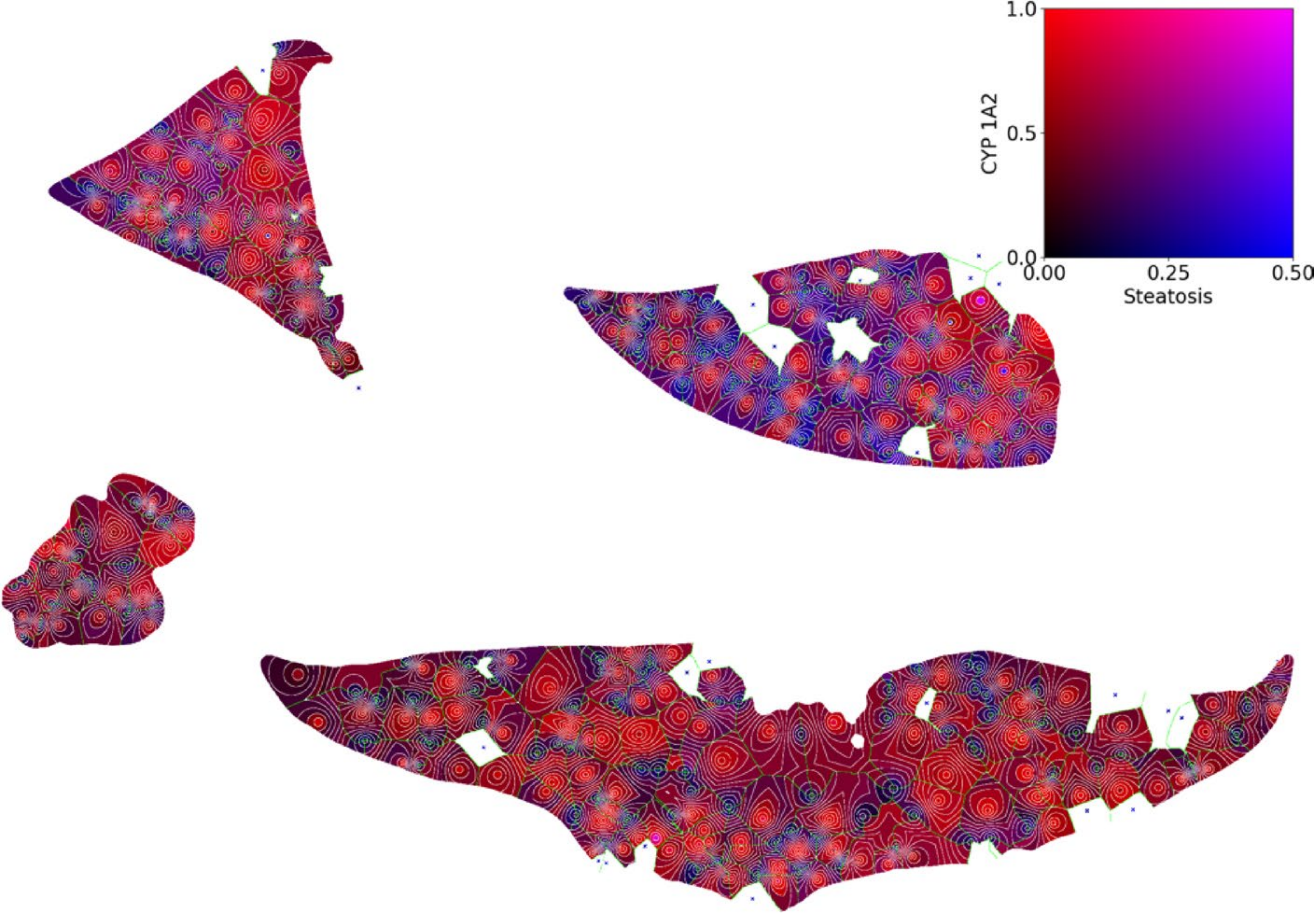
Overview of the heterogeneous dual parameter representation for all four liver lobes evaluated using the combined steatosis and CYP1A2 parameter map. The variable dual color intensity with areas in red and blue with others showing magenta indicates a heterogeneous signal pattern within the section. The missing lobules in the two liver lobes shown on the right side are caused by central veins without portal fields in their vicinity. These were probably missed due to the three-dimensional structure of the lobules and possible tissue distortions caused by cutting.

Figure 4 shows the dual channel color map of jointly quantified parameter pairs for a whole slide visualization showing the quantification of CYP1A2 and steatosis in one image. The visualization revealed wide blue areas around the portal vein indicating the presence of steatosis and the absence of CYP expression.

**Fig. 5:**
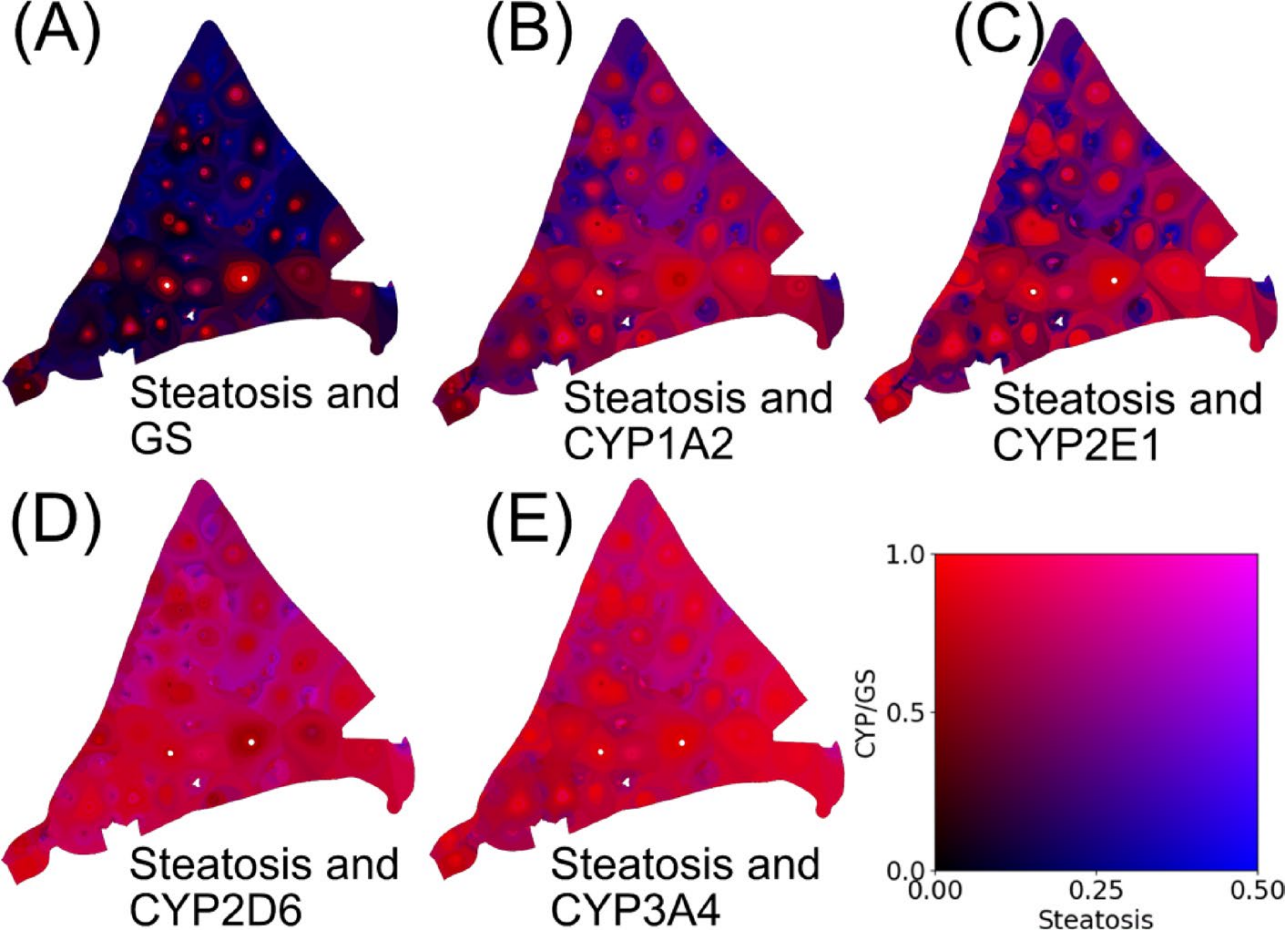
Combined parameter representation visualizing spatial distribution of steatosis combined with GS and each of the four CYPs in the same liver lobe. Blue indicates the presence of steatosis and absence of CYP expression, whereas red indicates the presence of GS or the respective CYP enzyme and absence of steatosis. Magenta indicates joint presence of steatosis and GS or CYP expression. (**A**) Visual representation of GS and fat droplet area fraction showing that GS is localized to the pericentral area, marked in red. In contrast, steatosis is localized mainly in the periportal area, marked in blue. (**B**,** C**) Heterogeneous distribution of steatosis and CYP expression revealing similar mixing patterns: CYP1A2 and CYP2E1 present periportal areas colored in blue showing steatosis but no CYP expression. Pericentral areas colored in red reveal no steatosis but strong CYP expression. In contrast, (**D**,** E**) Uniform distribution of CYP2D6 enzymes with a slight gradient CYP3A4 lt with steatotic areas in the periportal areas visualized in magenta, whereas pericentral areas with no or little steatosis but high CYP expression visualized predominantly in red.

The combined color maps for steatosis and all CYPs for a single patch are shown in Fig. 5. In contrast to CYP1A2, the small red rim around lobule centers represents the confined pericentral GS expression (see Fig. 5A). In contrast, combined visual representation of steatosis and CYP1A2, respectively, CYP2E1 highlighted the wider and more heterogeneous distribution of both CYP-enzymes, with periportal areas marked in blue indicating the presence of steatosis, and the absence of CYP expression. Furthermore, pericentral areas were colored in red of variable extent and distribution representing the more heterogeneous expression of the other CYP-enzymes (see Fig. 5B,C). When looking at all four liver lobes, as shown in Fig. 4, the parameter combination is even more heterogeneous.

**Fig. 6:**
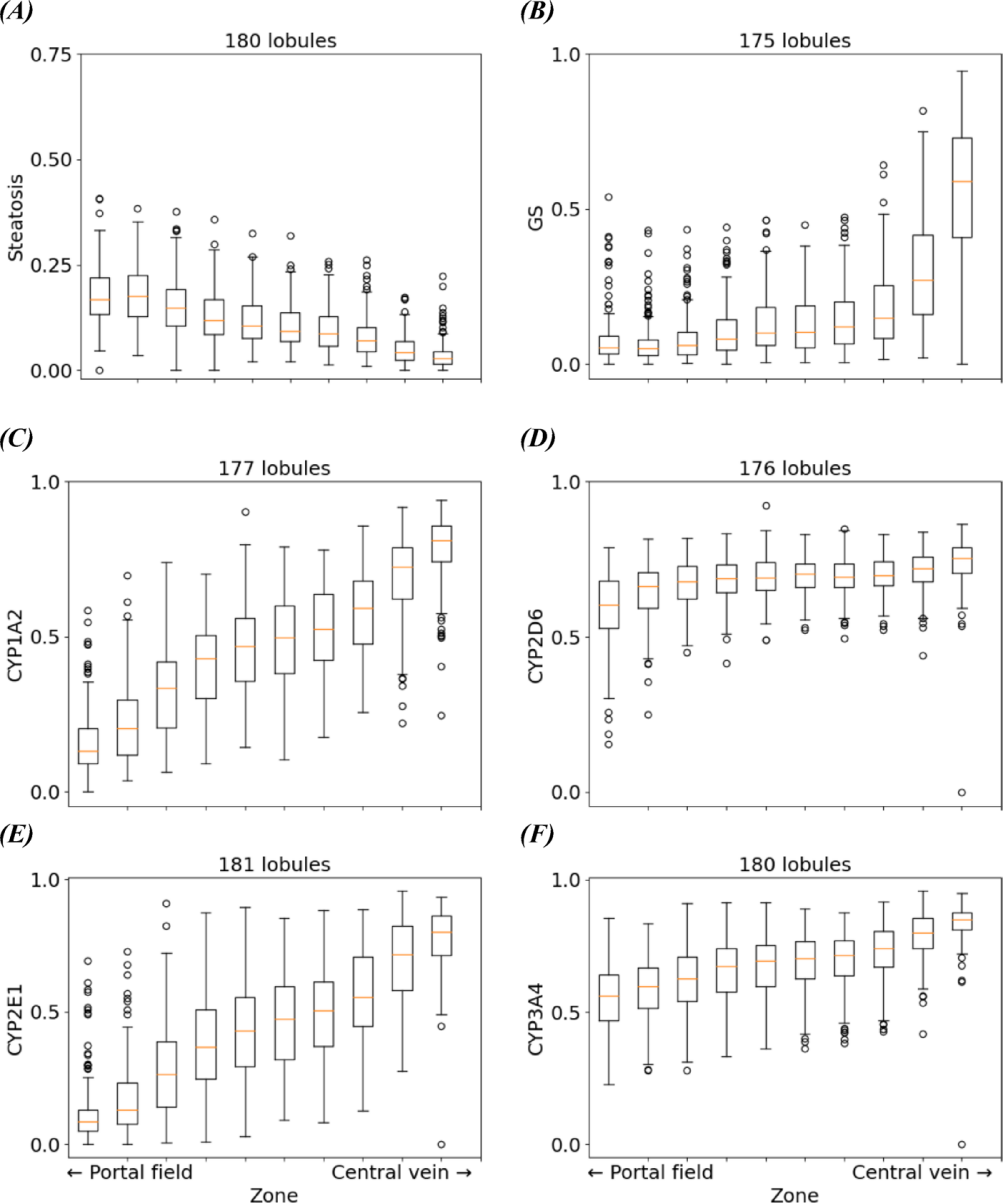
Box-and-whisker-plot quantifying the spatial distribution of parameters within the hepatic lobules of one moderately steatotic mouse liver along the sinusoidal porto-central axis. (**A**) Steatosis gently declines from the portal field to the central vein. (**B**) Intralobular zonal distribution of GS: GS is absent in portal zones, the steep gradient represents GS expression in pericentral zones Cytochrome P450 (CYP) enzymes show different patterns: (**C**) CYP1A2 is showing a steep increase over the zones, (**D**) no incline but rather uniform throughout the lobule for CYP2D, and (**E**) CYP2E1 only slowly inclines but is present throughout the lobule. (**F**) CYP3A4 shows a gentle incline from 0.5 to 0.8. The horizontal axis represents the portality index the periportal to pericentral. The vertical axis represents fat droplet area fraction and the ratio of positively stained over all tissue in each zone, respectively. The orange line indicates the median, the box shows the 1 st and 3rd quartiles, and the median whiskers extend up to 1.5 times the interquartile range. Data outside the whiskers range are displayed as circles.

Dual channel color map representation of steatosis and CYP2D6, respectively, CYP3A4 revealed another pattern. Periportal areas were marked in magenta indicating the joint presence of steatosis and CYP expression whereas the pericentral areas were marked in red representing the presence of CYP expression in the absence of steatosis (see Fig. 5D,E).

Complementing the geometrical visualization, the data distribution resulting from zonated quantification was displayed using box-and-whisker for single parameters and scatter plots for pairs of jointly quantified parameters.

As shown in Fig. 6 depicting the box-and-whisker plots for all observed parameters from one stack of images, the quantitative distribution of the different parameters within all lobules revealed a gentle negative gradient of the steatosis along the porto-central axis. In contrast, all marker proteins showed a positive gradient.

**Fig. 7:**
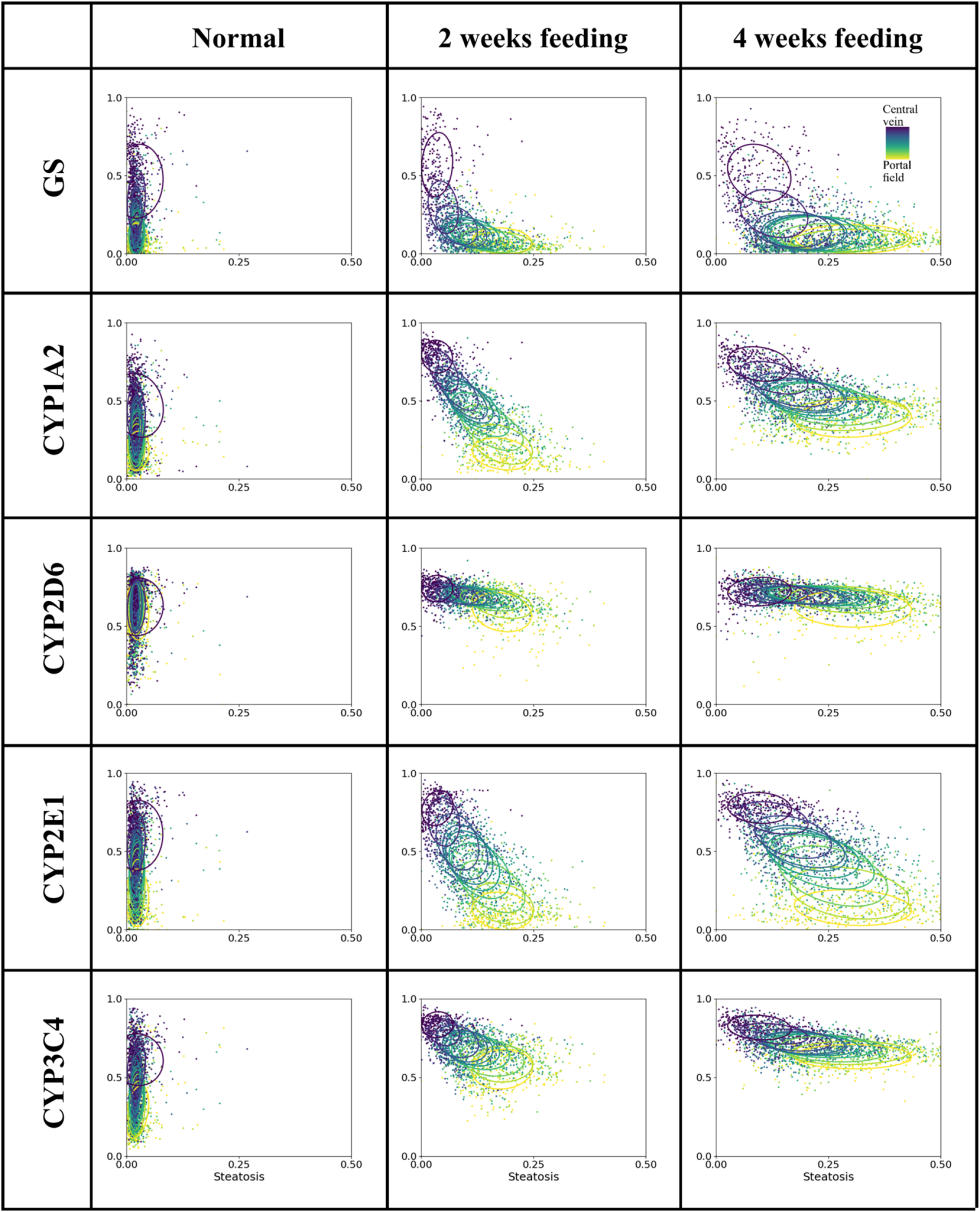
Scatter diagrams comparing the per-zone relation between the severity of steatosis and extent of zonal expression of GS and CYP enzymes showing three examples with different severity of steatosis. All diagrams show steatosis data (fat droplet area fraction) on the horizontal axis and ratio of positively stained tissue over all tissue on the vertical axis. The steatosis data is identical across the three mice. The color of dots and ellipses indicates the associated position along the sinusoidal porto-central axis from dark blue to yellow. The ellipses indicate mean and 2D standard deviation of the data for each zone across all lobules.

The scatter plots in Fig. 7 visualize the distribution of the data in joint zonated quantification. On the horizontal axis the severity of steatosis is displayed. On the vertical axis the ratio of positively stained tissue over all tissue is shown for all lobules under investigation. The color of dots and ellipses indicates the associated zone along the sinusoidal porto-central axis with yellow being closest to the portal field and dark blue closest to the pericentral vein. The ellipses visualize the mean and 2D single standard deviation limit of the data per zone. The extent of the ellipsoids represent the heterogeneity of the data, with wide ellipsoids representing large data variability. Hence, the relationship between the three parameters is indicated by the distribution and the color of the dots representing the zone.

We show and describe the scatter plots from three animals with different feeding scenarios to show the different patterns of signal distribution (see Fig. 7).

In all three livers, the expected pericentral expression of GS was observed. Combined with the absence of steatosis, this resulted in a narrow cluster of dark blue dots in the upper left quadrant and a quick drop of the subsequent ellipses. The absence of GS in the periportal zones is indicated by the lower position of the green and yellow ellipses. In the normal liver, no steatosis was seen, resulting in dots clustering on the left side of the plot. Despite increasing severity of steatosis, the light dark blue ellipsoid indicative of pericentral GS-expression remained in the upper left quadrant. In contrast, the yellow ellipsoid indicative of periportal steatosis became wider and shifted along the horizontal axis toward the right, with larger ellipses indicating higher interlobular heterogeneity for longer feeding intervals.

CYP1A2 and CYP2E1 demonstrated robust expression in proximity to the central vein, with a marked, linear decline in expression towards the portal region. This gradient is evident from the distribution of ellipses on the left side of the plot. In the four weeks feeding experiment, CYP1A2 expression extended into the periportal region, as indicated by the less steep gradient of ellipses, which did not approach zero. Similarly, CYP3A4 primarily displayed a pericentral expression pattern but was also present, albeit to a lesser extent, in the periportal region.

In contrast, CYP2D6 exhibited a relatively uniform expression throughout the lobules across all three feeding conditions, as evidenced by the nearly vertically aligned ellipses. Additionally, the variability of CYP2D6 expression was consistent across the lobule, as indicated by the comparable heights of the ellipses in each zone.

**Fig. 8:**
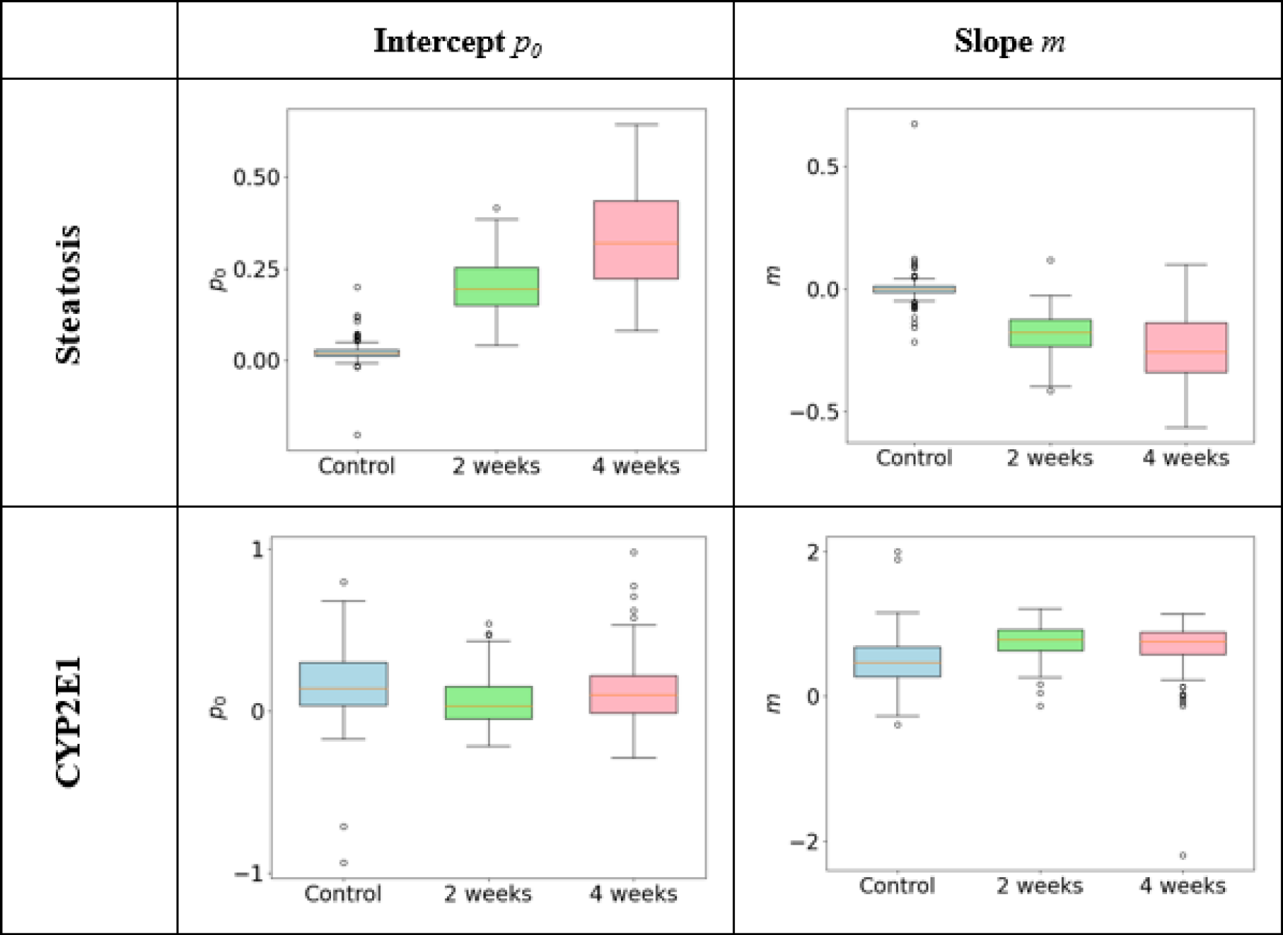
Quantifying and comparing linear fits to signal across lobules. Top: Steatosis in box-whisker-notation for intercept p_0_ (left) and slope m (right) of the linear model. An increase of the intercept and decrease if the slope corresponding to the different no, moderate and severe steatosis from right to left, was visible. Bottom: CYP2E1 was selected to demonstrate intercept and slope for one of the marker proteins. In both graphs, no clear trend was visible, corresponding to the expectation that CYP expression is not affected by steatosis. In the shown example case CYP2E1, more severe steatosis induced by a longer feeding period did not result in a visible change for slope m or intercept p_0_. A complete set of diagrams can be found in Figure S3 in the supplements.

The median intercept *p*_*0*_ and slope *m* analysis of steatosis characterizes the decrease of the steatosis levels along the position in the lobule (see Fig. 8). The control experiment shows a median slope of zero. The slope in the moderate case is negative with a median of *m* of −0.18 and in the severe case, the median slope is −0.26. The intercept in the three experiments suggests the extrapolated periportal maximum of the steatosis showing an increase from the healthy liver without steatosis to the liver with severe steatosis. For CYP2E1, ranges of the boxplots of slope *m* and intercept *p*_*0*_ for different steatosis levels largely overlap, indicating that there is no clear trend. This corresponds to the observation in Fig. 7 (CYP2E1, vertical axis) that the zonated CYP2E1 expression remains similar.

**Fig. 9:**
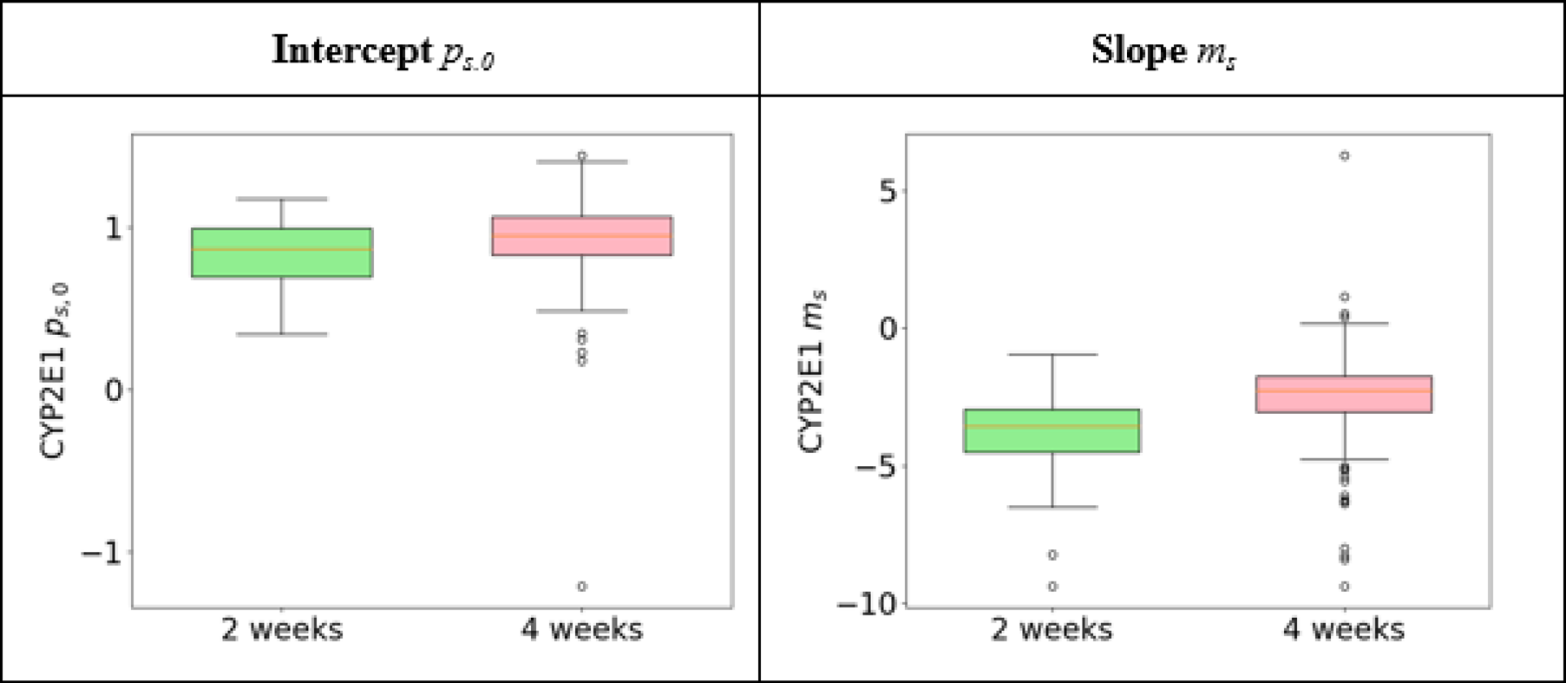
Quantifying and comparing linear fits for the expression of CYP2E1 presence in relation to the severity of steatosis using CYP2E1 as a showcase. Observed relationship between steatosis and CYP1E2, intercept with the p_s,0_-axis (left) and slope m_s_ (right). The median of the intercept is 0.87 for two and 0.95 for four weeks feeding, respectively. The slope has a median of −3.57 for two and − 2.26 for four weeks feeding, respectively. Slope as well as intercept show a largely overlapping distribution. The control group has been omitted because of the huge parameter range; a complete set of diagrams can be found in Figure S4 in the supplements.

To assess the relationship between steatosis and CYP2E1, we analyzed the slope and intercept values from a linear fit (see Fig. 9), focusing exclusively on the two conditions showing steatosis. The signal along the horizontal axis was essentially zero for the control liver without steatosis, resulting in an extensive range of slope and intercept values. Consequently, the median slope and intercept values were highly sensitive to minor fluctuations in the data, rendering this characterization unreliable (data shown in Supplementary Figure S4 for completeness). Comparisons presented in Fig. 9 quantitatively support the visual observations from Fig. 7 (see CYP2E1, middle and right panels), showing that the intercepts on the vertical axis were consistently near one, with no substantial differences. In contrast, the downward slopes varied by approximately a factor of two.

## Discussion

We presented a workflow to jointly evaluate multiple histological parameters based on an idealized zonal and lobular geometry by dividing the porto-central axis arbitrarily into twelve zones. The workflow was established using WSI from livers with periportal steatosis stained for GS and four different CYP enzymes. We observed in selected samples with and without periportal macrovesicular steatosis that the zonal distribution of GS and CYP was similar, suggesting that extent and zonal distribution was not affected by the presence of macrovesicular steatosis.

This agrees well with Lindros et al. 1997^6^ and our previous work^9^ where all hepatic CYP enzymes were found to be expressed in the pericentral region of the hepatic lobule of different species, but with noticeable differences between the species, except for CYP2D6 expressed throughout the entire lobule.

However, the use of our algorithm will allow the extension of previous observations. Previously, quantification of steatosis and biomarker expression were not co-visualized. Joint quantification, statistical representation and visualization of the results render different expression patterns more comparable.

Our methodology allows the investigation of parameters from multiple histological measurements on a lobule and zone level by registering adjacent differentially stained histological sections and identifying corresponding lobules and zones.

Visualization is based on multi-parametric color coding (see Figs. 4 and 5) or achieved via scatter plots showing relations of parameter distributions in lobules and zones (see Fig. 7). Combining all available information for individual zones in individual lobules results in the graphical and statistical representation of quantitative morphological and functional information in terms of zonated quantification of the underlying expression pattern.

The characterization of zonation trends using curve fitting can provide further insights into underlying biological mechanisms. We assumed the simple case of a linear model parameterized by intercept and slope, which visually matches the steatosis and CYP data, but does not approximate the GS data well. However, other curves can be used instead of a linear fit, e.g., exponential or sigmoid, matching knowledge or hypotheses about the underlying biological mechanisms.

The presented methodology is modular and the tools for registration, parameter evaluation, and computation of lobules and zones can be modified and replaced independently in the workflow. With multiple parameters extracted from the same image, image registration and potentially transferring annotation coordinates can be skipped. For instance, steatosis could be detected in CYP staining, or PF/CV locations could be detected automatically^26^ or in other stainings.

As one building block of the methodology presented here, we implemented a new method to automatically quantify the presence of positively stained biomarkers (here, CYP proteins in IHC staining using pixel classification). Based on expectation maximization per WSI, this approach is robust against color variations between sections and images but not to local variations within a WSI. It only estimates the actual distribution of positive signals and may produce unsatisfactory results on examples with less optimal staining. For this reason, we selected the data set based on technical criteria.

Other methods for CYP detection employed color deconvolution^21^. However, both approaches can only quantify the presence of positive signals representing biomarker expression in terms of positive signals, in our case, brown pixels. This does not allow an absolute quantification of the amount of CYP present, nor does it reflect the activity of the protein. If this information is needed, complementary assays must be performed, knowing that e.g. current activity assays require tissue homogenization, thereby not allowing any zonated quantification. However, a combination of spatially resolved quantification of expression and a global activity assay could still help to better estimate the lobular functionality.

### Limitations

Limitations of determining lobules based on PF and CV positions have been discussed by Schwen et al. 2016^17^. Transforming the vessel positions and creating new lobules and zone masks instead of transforming each pixel leads to slightly differing masks for the stained slides. It must be investigated whether these differences reflect the lobular structure more accurately than a pixel-based transformation used in Albadry et al. 2024^9^. The registration direction may also be of more importance when transforming the image instead of vessel positions. It requires investigating the potential impact on the results if one of the CYP-stained sections is chosen as a reference and the H&E-stained sections are transformed.

The distance between the sections may have an impact on their similarity and, subsequently, on the image registration quality. In our case, this was avoided by using sections of 3 μm thickness. Increasing the thickness of the sections or omitting a technically inappropriate section might render transformation less reliable.

The underlying registration method can introduce errors if not properly configured. With a non-rigid registration, the images may be warped locally. Therefore, it is important to assess the images after registration to identify erroneous deformations and their impact on the identification of lobules and zones.

The image registration method employed here was previously validated in, e.g., the ANHIR and ACROBAT challenges^33,34^. We checked the accuracy of the image registration by visually assessing shifts between neighboring slide images. A more comprehensive validation will, on the one hand, require manually annotated landmarks to measure test point errors^35^, or generating artificial images, deforming them and measuring the distance between the ground truth deformation and the registration result^36,37^. On the other hand, the impact along the entire workflow will need to be assessed, comparing results of the zonated quantification or downstream tasks to a suitable ground truth or reference result. Pending a comprehensive validation of the methods for joint zonated quantification presented in this study, our example results, albeit plausible and aligning with previous findings, should not be generalized.

The central limitations of this study are twofold: A repeatability analysis of the jointly zonated quantification results is missing and will need to be performed using multiple adjacent stacks of slides from the same liver. Moreover, the descriptive proof-of-concept results from single livers do not permit generalizable biological conclusions.

### Perspectives

As a subsequent step, the algorithm presented herein should be employed to investigate a biological question in multiple mice per group. One could, e.g., examine the influence of zonated steatosis severity on the zonated expression patterns of CYP enzymes and their resulting functions. Such investigations would benefit from the incorporation of advanced statistical methodologies, including super-plots, to rigorously assess both intra- and inter-sample variability, thereby enhancing the reproducibility and interpretability of the observed biological phenomena^38^. Furthermore, the determination of the used parameters could be improved or complemented with additional biological information. As an example, the fat quantification could be extended to include microvesicular steatosis. As mentioned above, the results of zonated CYP quantification should be related to protein quantification via western blot and to the results of activity assays to reach a complete and comprehensive data collection for better understanding of the underlying biological mechanisms. The additional linear modelling of the distribution of steatosis and CYP over the radius of the lobule might prove valuable when setting up realistic simulation models for liver lobules in pharmacokinetic simulations.

The zonated quantification results are spatially autocorrelated, which could be investigated further. It can be quantified using, e.g., global and local Moran’s I^39,40^ to further characterize the spatial patterns of jointly quantified parameters and the corresponding biological phenomena. However, quantifying autocorrelation requires considering details such as choosing a distance metric (e.g., Euclidean or neighborhood-based) suitable for the underlying biological mechanisms. Statistical hypothesis testing involving data per individual lobule also needs to compensate for spatial autocorrelation. Hypothesis testing involving the multi-dimensional joint zonated quantification results could employ a permutation test^41^ using the energy statistic^42^ with a permutation strategy adapted for autocorrelation.

On the image analysis side, it might be possible to extract multiple parameters from the same image, e.g., by quantifying steatosis from fat vacuoles in CYP-stained sections or using immunofluorescence imaging with multiple fluorophores for more than one parameter. This would obviate the need for image registration but still would benefit from a zonated quantification of multiple parameters visible in the same slide.

The detection of vessels could be automated^9,26^. However, irregularities in the anatomy can lead to errors in lobule detection. Merging adjacent central veins or disregarding those in very close proximity might prevent such errors in lobule detection. Similarly, artifacts due to lobules detected across tissue gaps could be avoided. On the one hand, automatic tissue fragment separation and masking would be beneficial for slides containing multiple pieces of tissue. On the other hand, a topology-aware watershed transformation could avoid lobules across tissue indentations.

The method of merging colors to display multiple parameters could be applied to another color model (cyan-magenta-yellow or hue-saturation-value). This approach would allow the comparison of three parameters in one image. The scatter diagrams are the basis for cluster analysis of parameter distribution in the zones, possibly allowing the identification of heterogeneity in zonal parameter distributions within lobules located in different regions of the liver. This might be linked to an autocorrelation analysis and would address this from two differing perspectives.

Importantly, the quantification results can be used to more accurately parameterize computational simulations of physiological processes in the lobules and zones. Doing so could help to model, for example, the impact of a morphological disorder such as fibrosis or cirrhosis on a functional parameter.

## Conclusion

Establishing joint zonated quantification of CYP expression in a steatotic liver as a showcase, we visualized differences in the relationship between macrovesicular steatosis, GS and four CYP enzymes in the selected tissue samples. However, visualization of multiple parameters requires reliable image registration processes to avoid errors due to non-rigid transformations. The proposed workflow opens new possibilities to quantify the zonated expression of marker proteins with respect to the underlying zonated pathology on a lobule and zone basis.

## Supplementary Information

Below is the link to the electronic supplementary material.

Supplementary Material 1

## Data Availability

The datasets generated and/or analysed during the current study are available in the Zenodo repository, [https://zenodo.org/doi/10.5281/zenodo.12818566]^43^ and FairdomHub [https://doi.org/10.15490/fairdomhub.1.study.1070.1].
