# Supplementary material for: Joint zonated quantification of multiple parameters in hepatic lobules
Source: Sci Rep. 2026 May 14;16:15207. doi: 10.1038/s41598-026-46721-5 (PMC13179316; doi:10.1038/s41598-026-46721-5)
Supplement: Supplementary file 1 — Supplementary Material 1 [file 41598_2026_46721_MOESM1_ESM.docx]

# **Supplements**

| ***(A)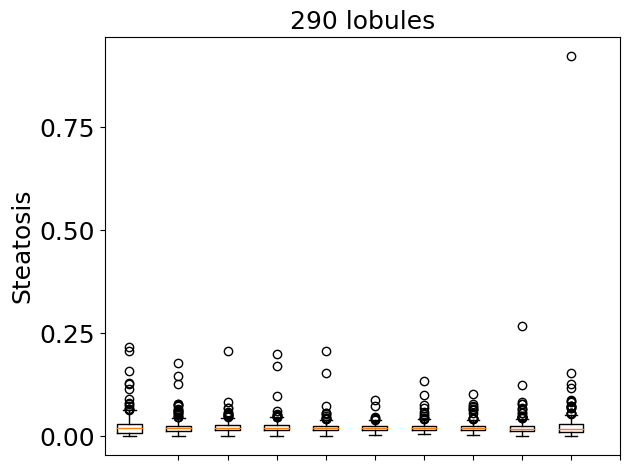*** | ***(B)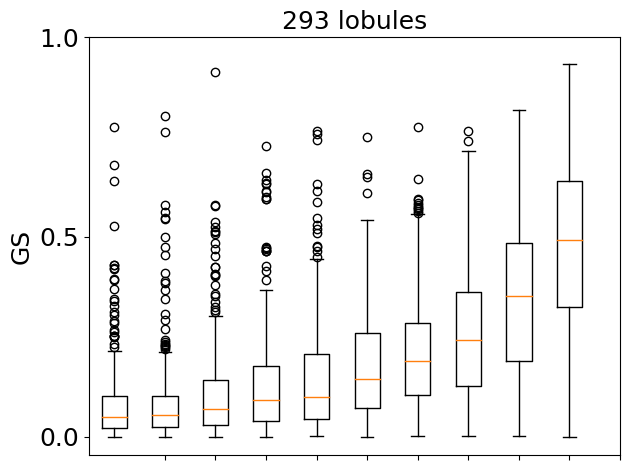*** |
| --- | --- |
| ***(C)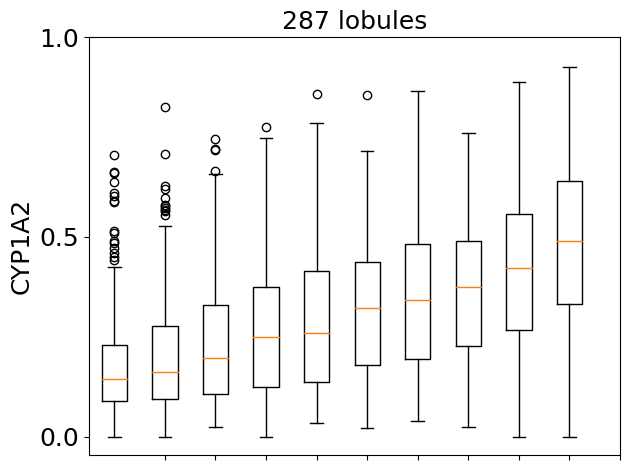*** | ***(D)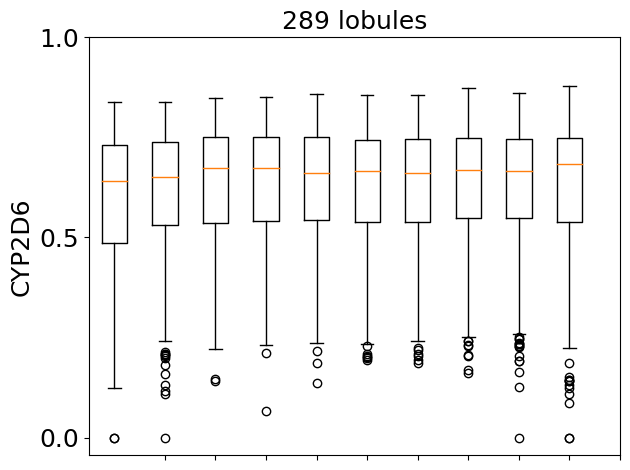*** |
| ***(E)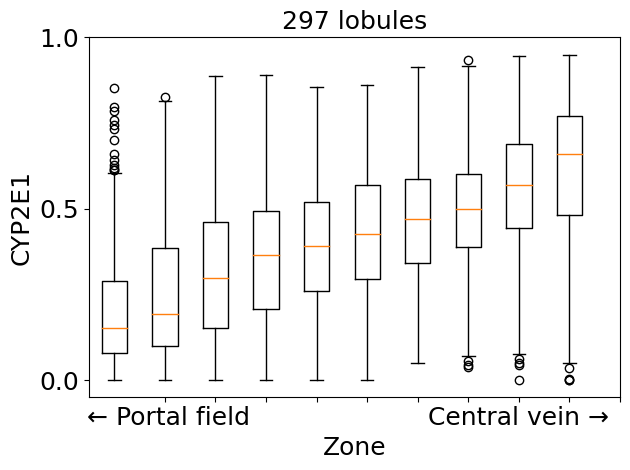*** | ***(F)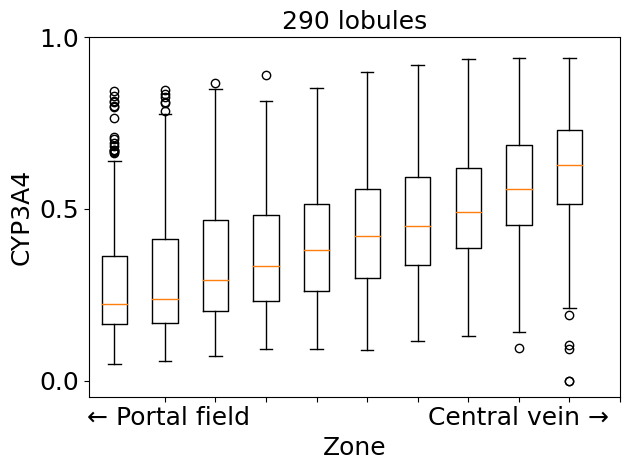*** |

***Figure S1: Box-whisker plot of the spatial distribution of parameters within the hepatic lobules for the control group. (A)****There is no steatosis in the lobules.* ***(B)*** *Intralobular zonal distribution of GS: GS is absent in zones 1 to 6 and shows a gradual increase of GS expression in zones 7 to 11.* ***(C)*** *CYP1A2 is predominantly expressed in the pericentral region, (****D)*** *CYP2D6 shows no gradient, and* ***(E, F)*** *CYP2E1 and CYP3A4 also show an increase from the periportal to the pericentral region. The horizontal axis represents the portality index arbitrarily divided into 12 zones with one and twelve omitted. The vertical axis represents fat droplet area fraction and the ratio of positively stained over all tissue in each zone, respectively. The orange line indicates the median, the box shows the 1st and 3rd quartiles, and the median whiskers extend up to 1.5 times the interquartile range. Data outside the whiskers range are displayed as circles.*

| ***(A)****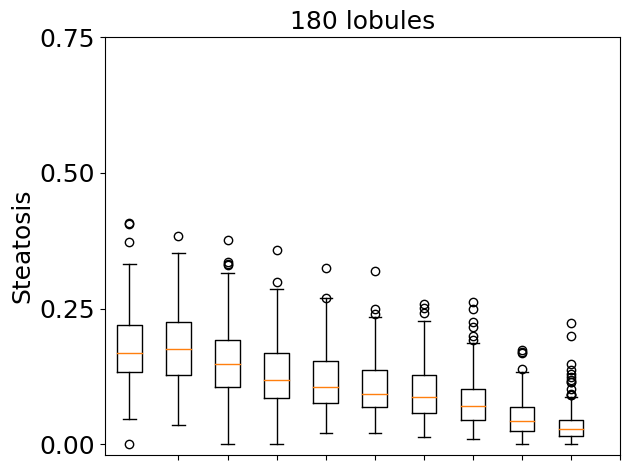* | ***(B)****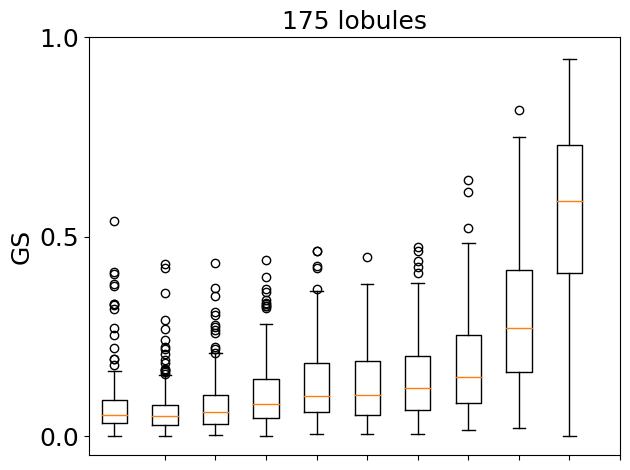* |
| --- | --- |
| ***(C)***  *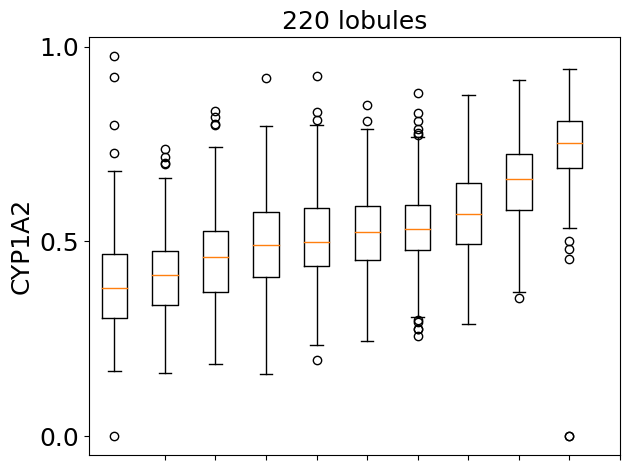* | ***(D)****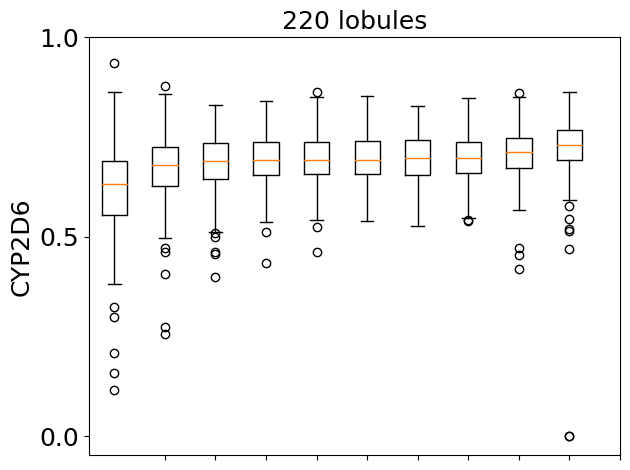* |
| ***(E)****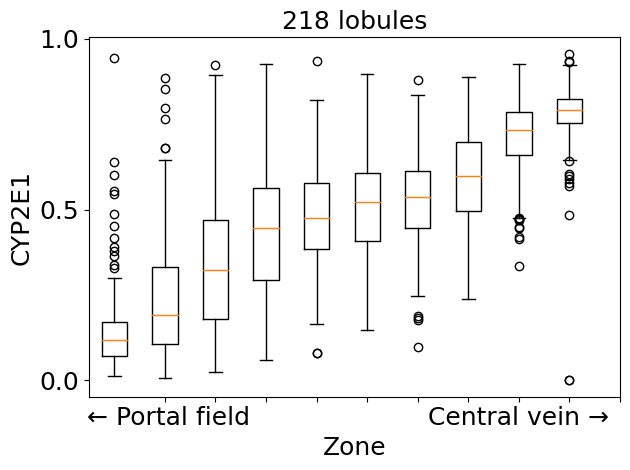* | ***(F)****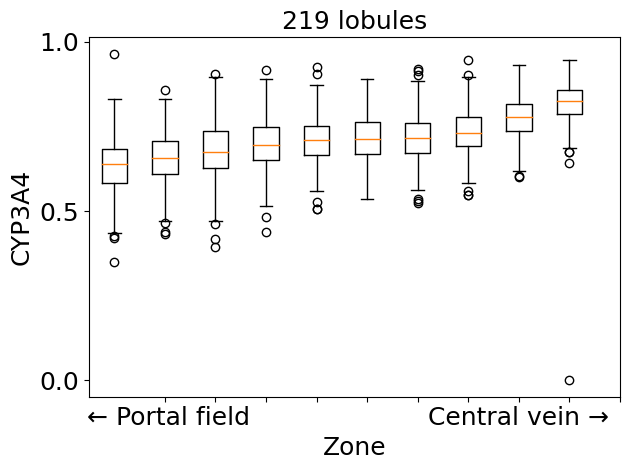* |

***Figure S2: Box-whisker plot of the spatial distribution of parameters within the hepatic lobules the control mouse for 4 weeks feeding mouse (A)*** *Steatosis shows a steeper decline from portal to central vein than in Figure 5.* ***(B)*** *GS is absent in zone 1-8 and shows a steep gradient in zone 9-11, slightly steeper than in Figure 5. Cytochrome P450 enzymes:* ***(C)*** *CYP1A2 is predominantly expressed in the pericentral region, but present throughout the entire lobules, (****D)*** *CYP2D6 shows no gradients but larger ranges of values throughout the slide in the zones closest to the portal field,* and **(E)** CYP2E1 shows an increase from periportal to pericentral region, **(F)** *CYP3A4 is present throughout the entire lobules with a gentle incline towards the pericentral region.*

|  | **Intercept *p_0_*** | ***Slope m*** |
| --- | --- | --- |
| **Steatosis** | *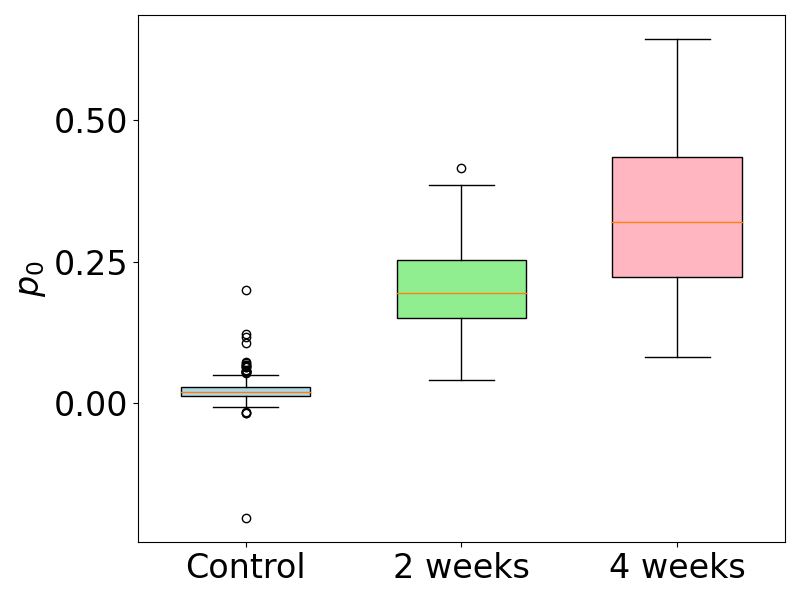* | *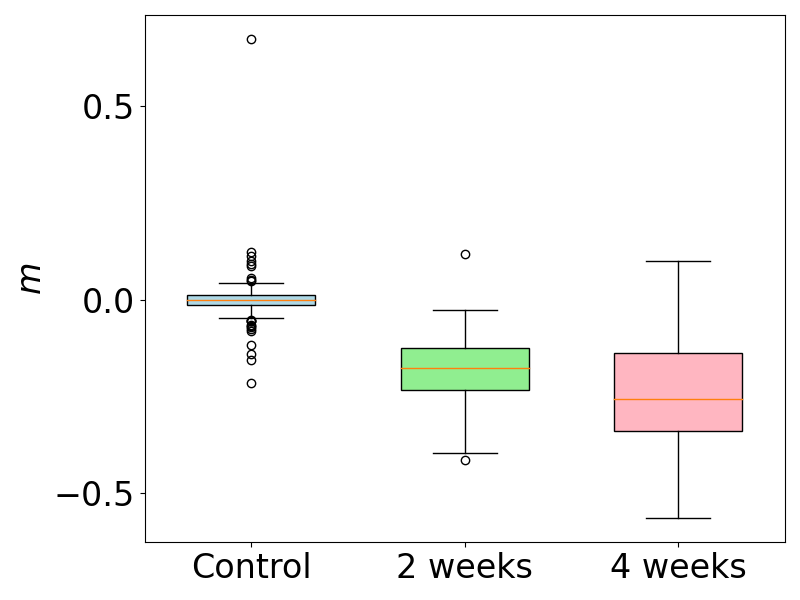* |
| **GS** | *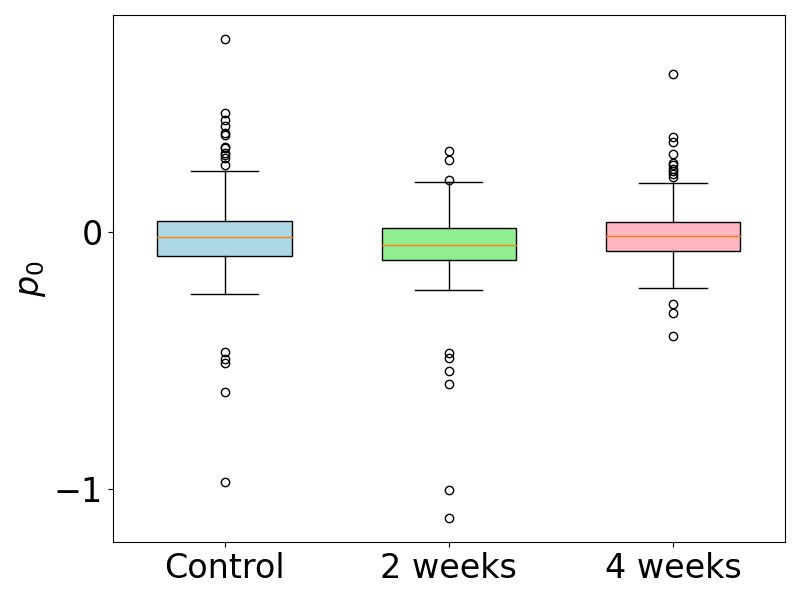* | *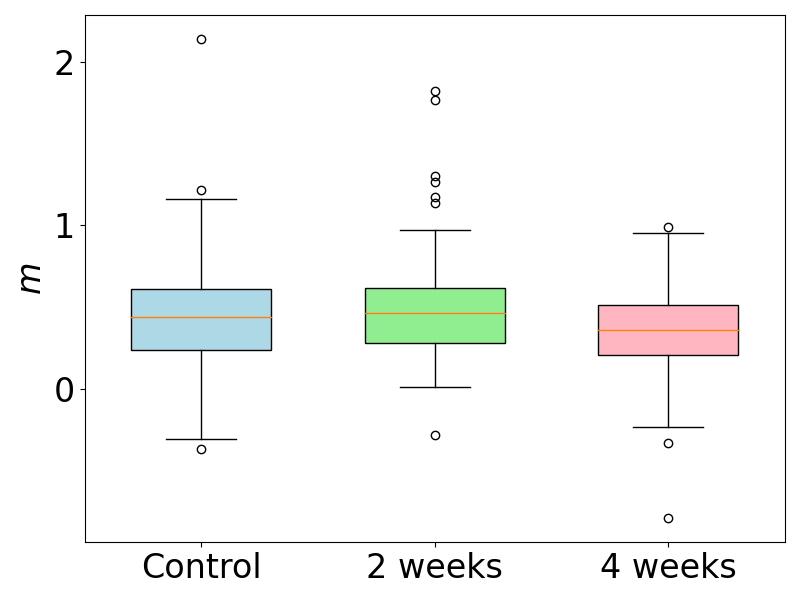* |
| **CYP1A2** | *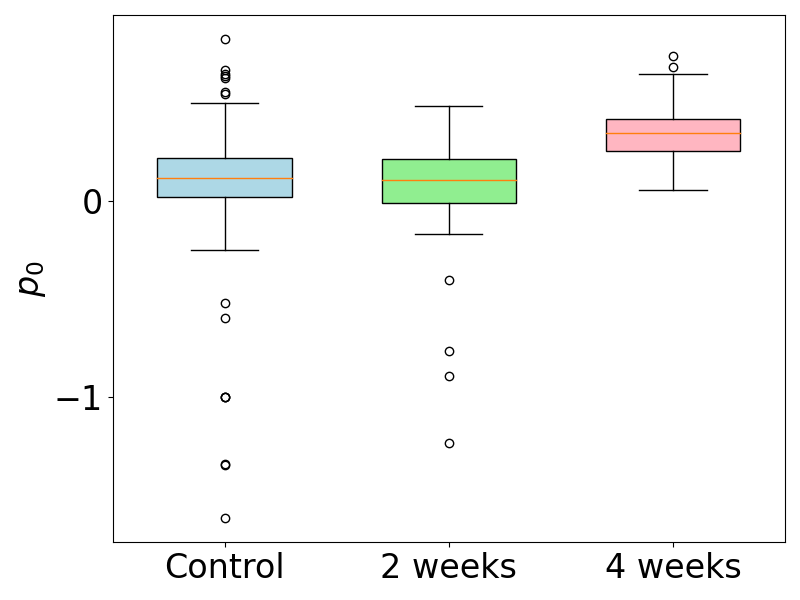* | *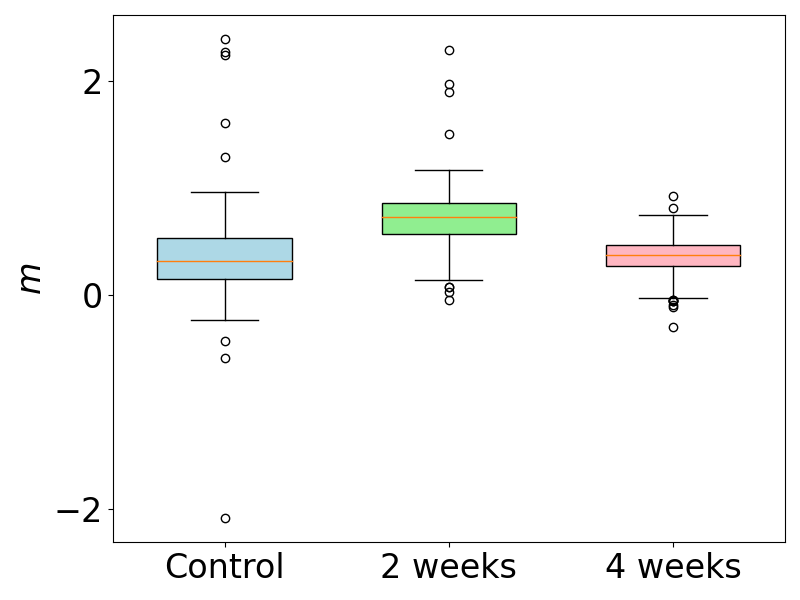* |
| **CYP2D6** | *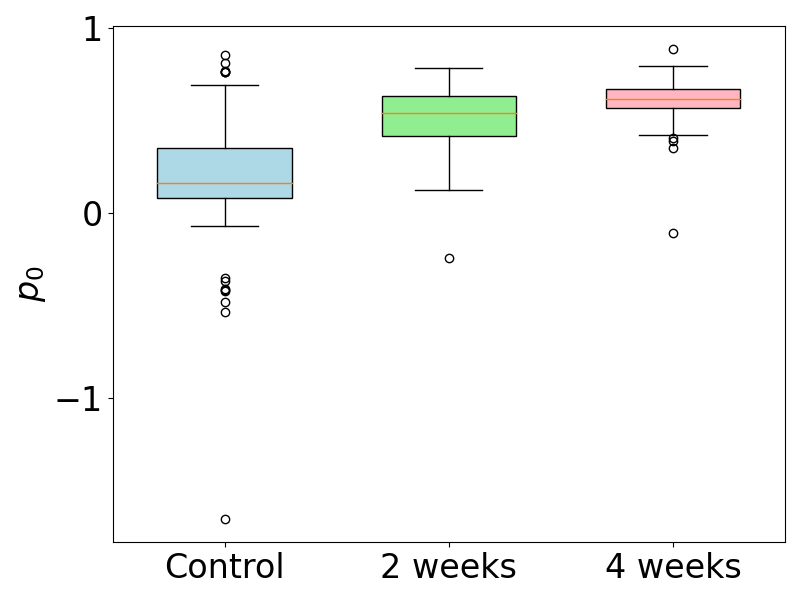* | *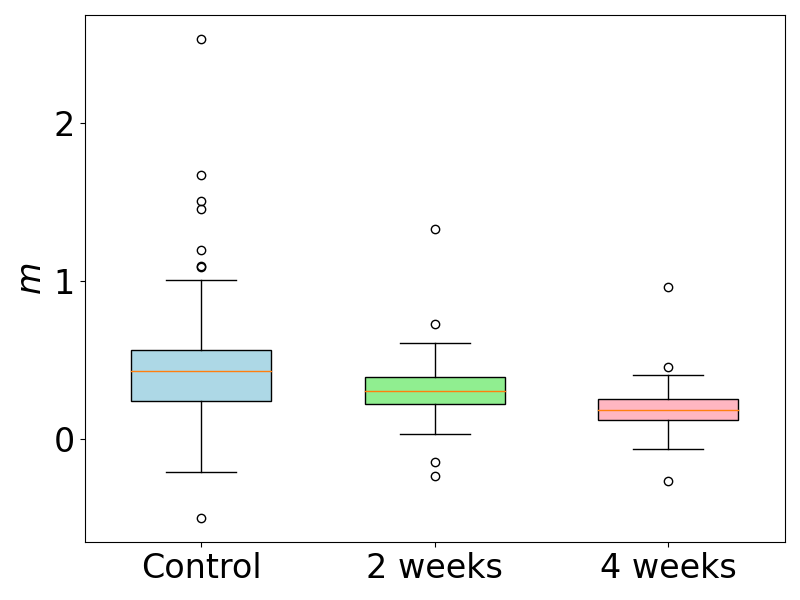* |
| **CYP2E1** | *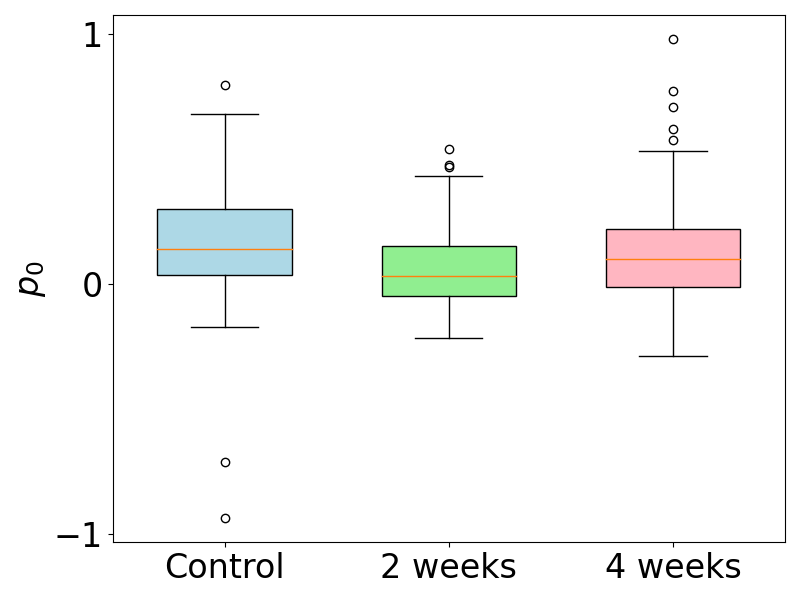* | *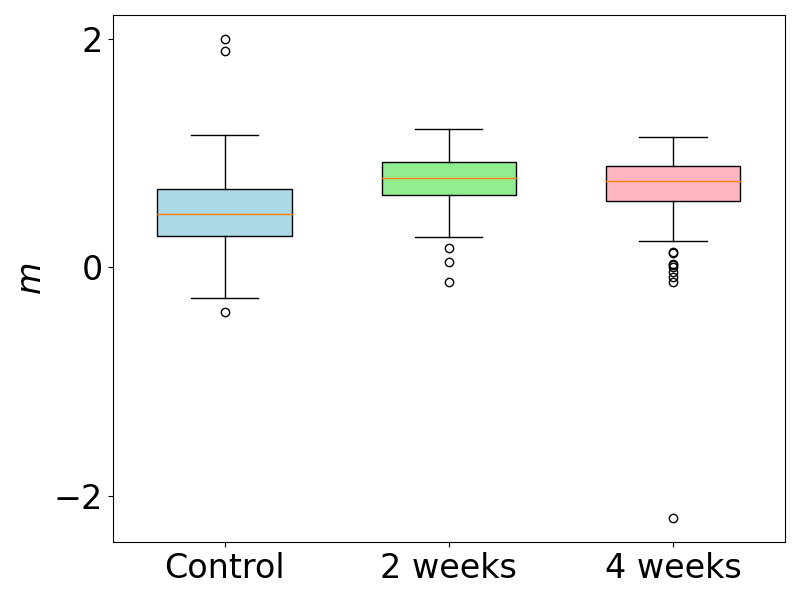* |
| **CYP3A4** | *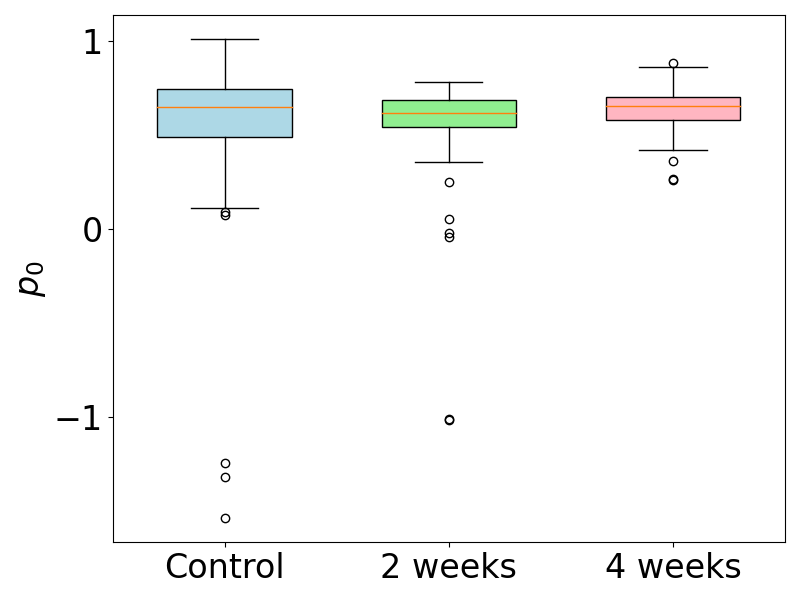* | *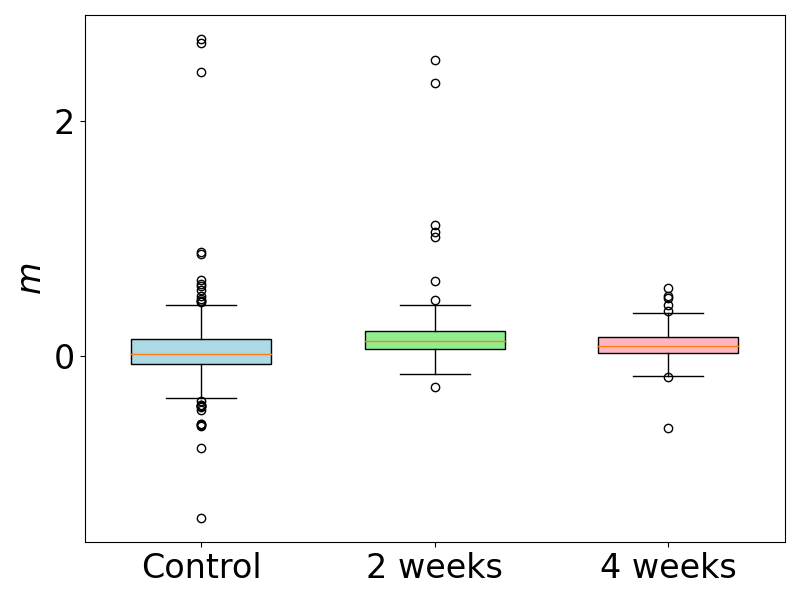* |

***Figure S3: Quantifying and comparing linear fits to signal across lobules. CYP3A4, CYP2E1:*** *For these enzymes, p_0_ remains constant across the three experimental conditions (Healthy control, moderate and severe steatosis induced by feeding mice for 2 respectively 4 weeks indicating no change in the pericentral presence. The slope m is about zero, indicating a constant and unchanged distribution over the lobule.* ***CYP2D6:*** *p_0_ increases with prolonged feeding, proposing an increase in initial pericentral presence. The slope m is slightly decreasing with feeding duration, but the overlapping quartiles indicate no notable change.* ***CYP1A2:*** *p_0_ shows a low initial periportal presence for control and two weeks of feeding while a slight increase is visible for four weeks feeding. The slope m is slightly positive and increasing from control to 2 weeks. 4 weeks is similar to the control group.* ***GS:*** *a p_0_ of about zero for all groups indicates no initial periportal presence, and slope m is also unchanged over the feeding situations. Steatosis was already shown and described in Figure 8.*

*.*

|  | **Intercept** *p_s,0_* | **Slope** *m* |
| --- | --- | --- |
| **Steatosis vs GS** | *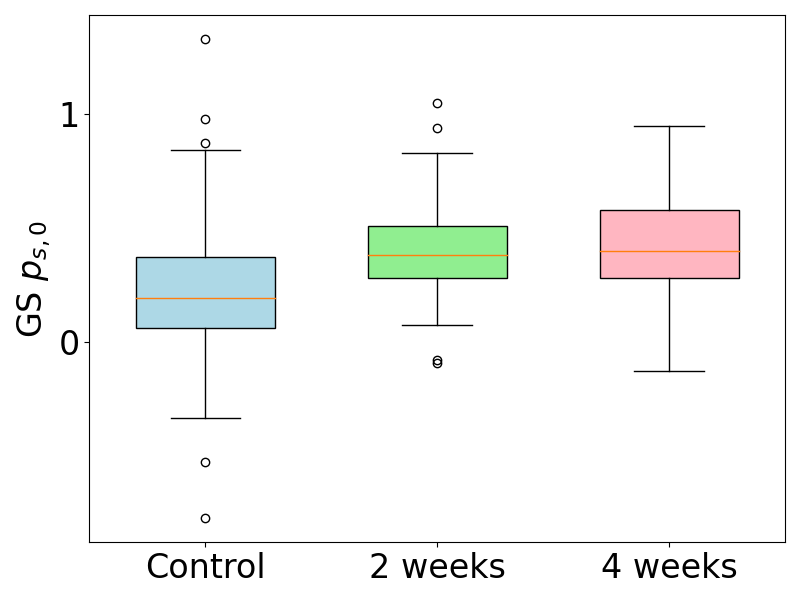* | *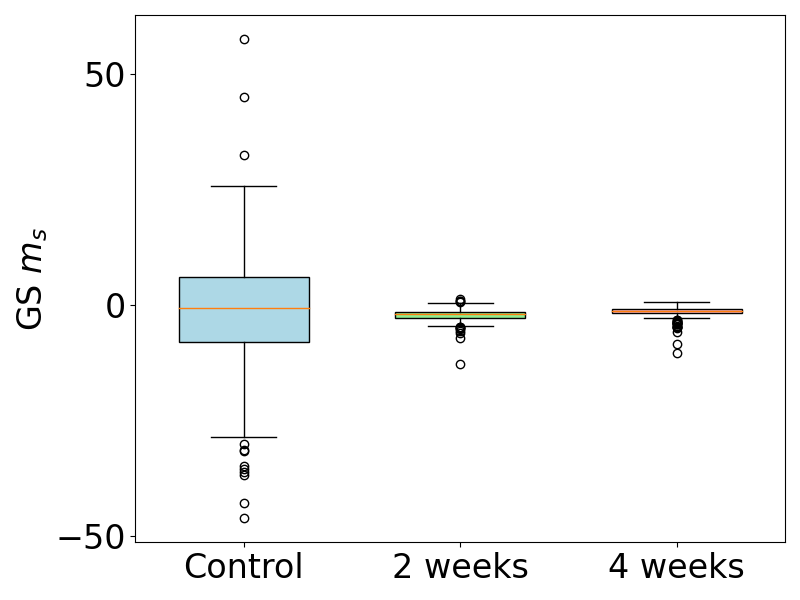* |
| **Steatosis vs CYP1A2** | *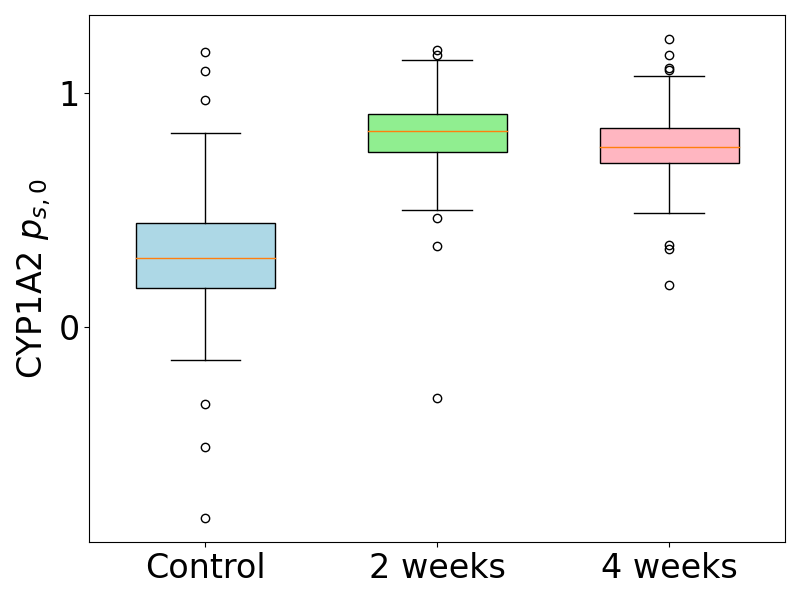* | *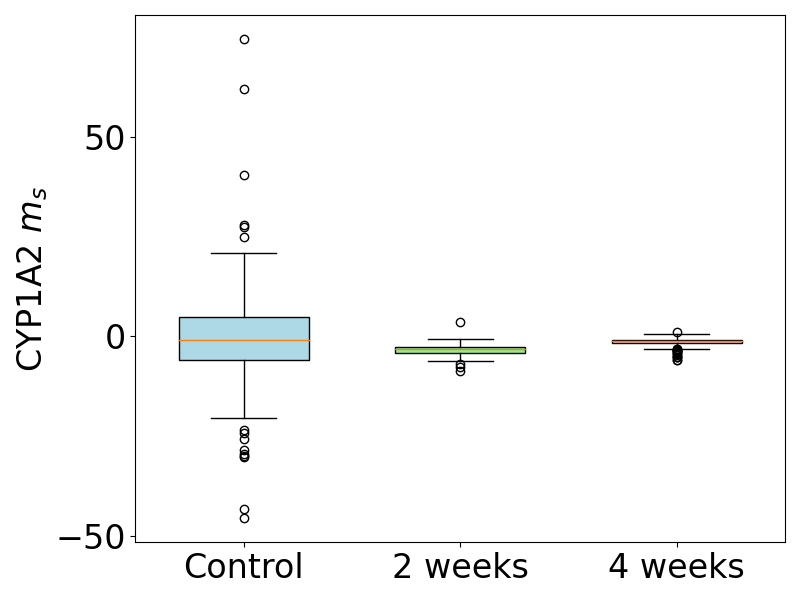* |
| **Steatosis vs CYP2D6** | *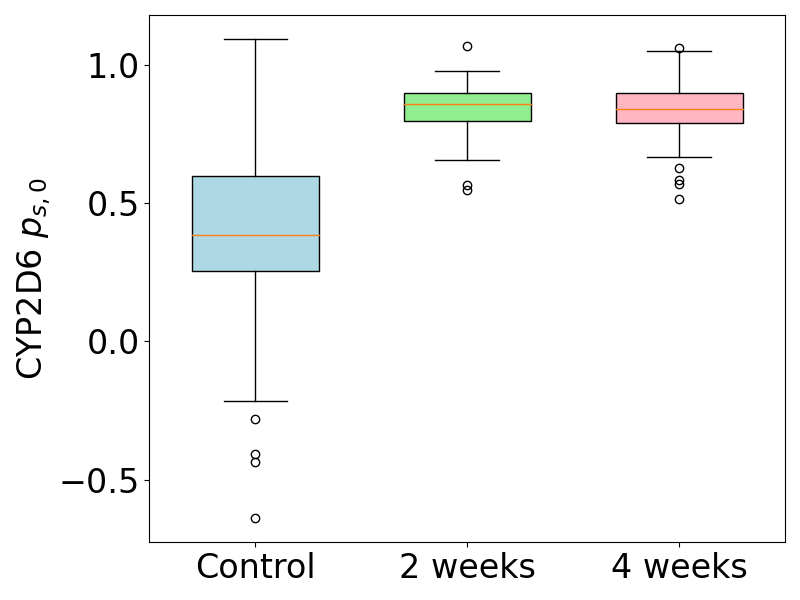* | *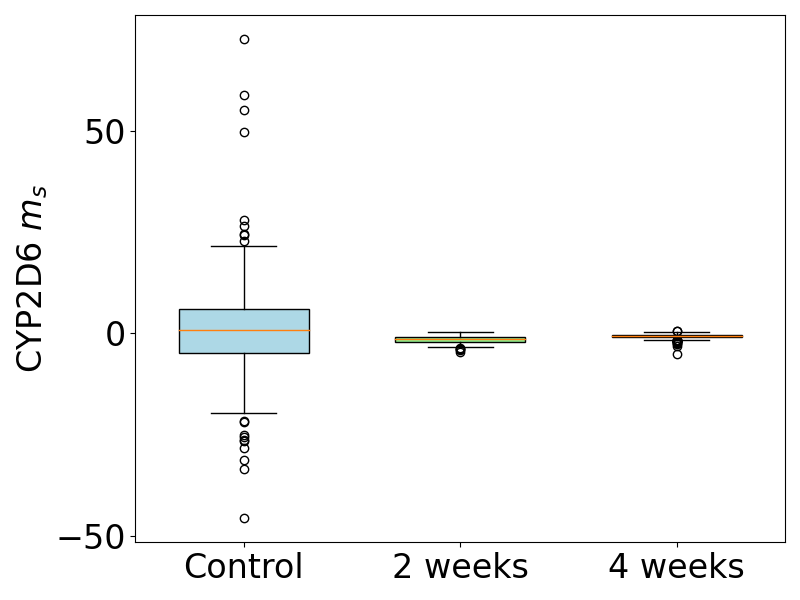* |
| **Steatosis vs CYP1E1** | *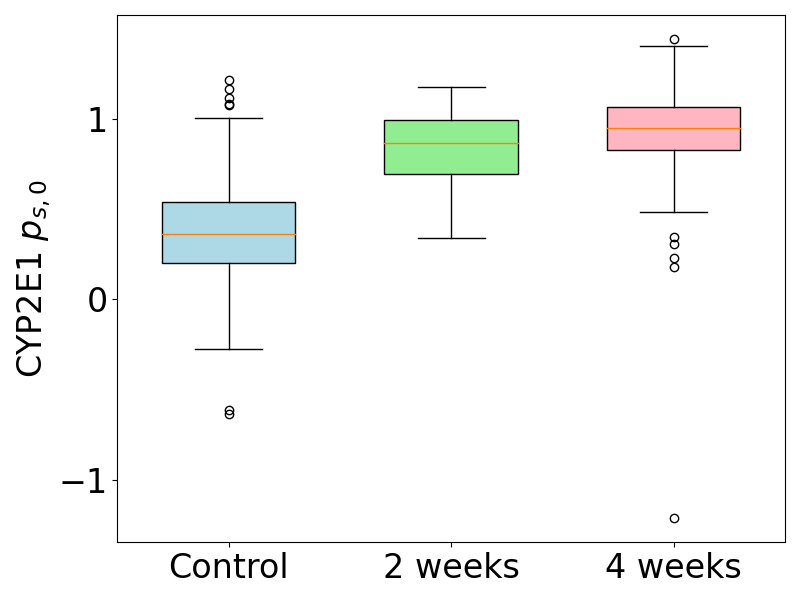* | *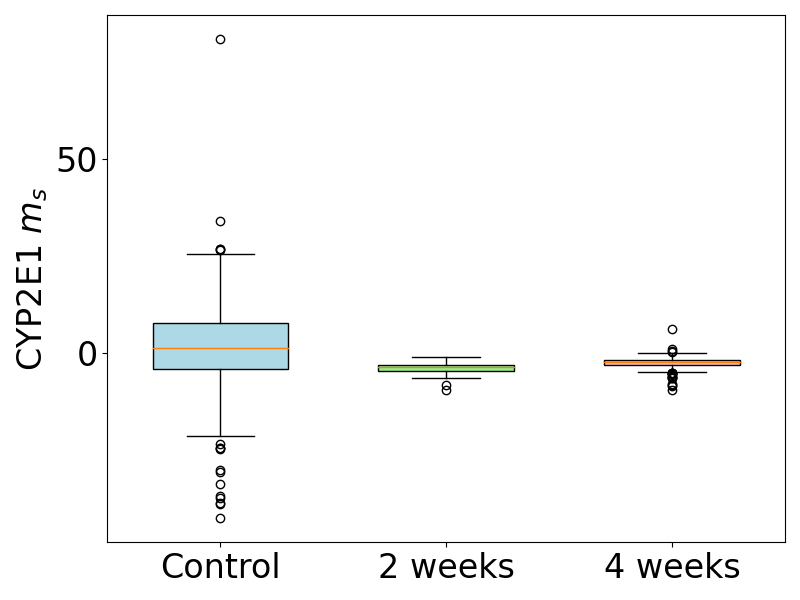* |
| **Steatosis vs CYP3A4** | *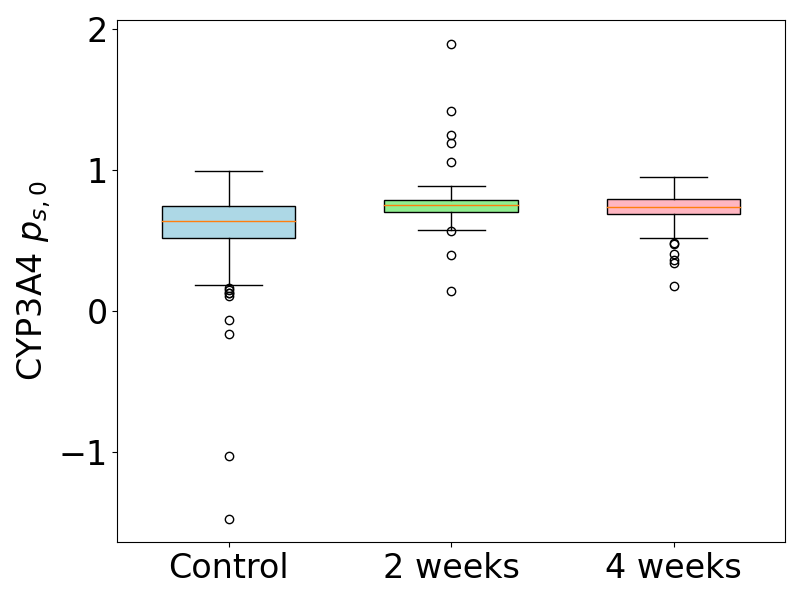* | *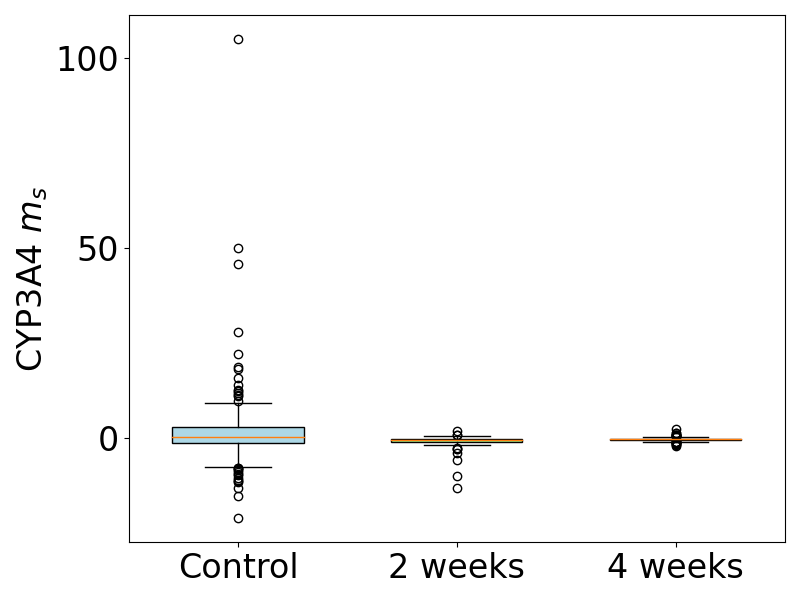* |

***Figure S4: Quantifying and comparing linear fits CYP/GS presence in relation to steatosis severity.*** *Observed relationship between steatosis and CYP/GS, intercept with the p_s,0_-axis (left) and slope m_s_ (right). The intercept p_s,0_ is zero for CYP3A4 in all feeding situations. All other CYPS and GS show a lower p_s,0_ for the control group and comparable ones for two and four weeks of feeding. The diagram show also includes the control group which showed a highly variable slope m_s_ making it difficult to compare the slopes for two and four weeks feeding.*

***Table S1:*** ***Mean and standard deviation of the slope m of the lobule based linear model fitting across the lobule.*** *The increase of the slope in steatosis is clearly visible by an increase from zero to -0.18 for two weeks and -0.24 for four weeks feeding, respectively. The trend for the slopes for the enzymes is less clear for the other enzymes and shows no clear trend from control to four weeks.*

| Mouse | steatosis *m* | GS *m* | CYP1A2 *m* | CYP3A4 *m* | CYP2E1 *m* | CYP2D6 *m* |
| --- | --- | --- | --- | --- | --- | --- |
| Control | -0.00±0.05 | 0.44±0.29 | 0.35±0.38 | 0.05±0.35 | 0.47±0.33 | 0.43±0.29 |
| 2 Weeks | -0.18±0.08 | 0.47±0.28 | 0.73±0.29 | 0.18±0.29 | 0.76±0.22 | 0.32±0.15 |
| 4 Weeks | -0.24±0.14 | 0.36±0.23 | 0.37±0.18 | 0.09±0.13 | 0.70±0.31 | 0.19±0.11 |

***Table S2:*** ***Mean and standard deviation of the intercept p_0_ of the lobule based linear model fitting across lobules.*** *Again intercept p_0_ of steatosis is clearly increased over the three groups. A similar increase is visible for CYP2D6 but only when comparing control with the other groups.*

| Mouse | steatosis *p_0_* | GS *p_0_* | CYP1A2 *p_0_* | CYP3A4 *p_0_* | CYP2E1 *p_0_* | CYP2D6 *p*_0_ |
| --- | --- | --- | --- | --- | --- | --- |
| Control | 0.02±0.02 | -0.02±0.15 | 0.11±0.26 | 0.59±0.27 | 0.17±0.20 | 0.21±0.24 |
| 2 Weeks | 0.21±0.07 | -0.06±0.16 | 0.09±0.20 | 0.59±0.21 | 0.06±0.15 | 0.52±0.14 |
| 4 Weeks | 0.33±0.13 | -0.01±0.11 | 0.35±0.12 | 0.64±0.09 | 0.12±0.18 | 0.62±0.10 |

***Table S3:*** ***Mean and standard deviation of the slope m_S_ of the lobule based linear model fitting for steatosis vs CYP/GS.*** *As visible in Figure S4, the distribution of slopes m_s_ for the control group shows very strong variations caused by the very small interval of steatosis value. In case of the other groups the change from two to four weeks feeding usually remains with the range of the standard deviations.*

| Mouse | GS *m_s_* | CYP1A2 *m_s_* | CYP3A4 *m_s_* | CYP2E1 *m_s_* | CYP2D6 *m_s_* |
| --- | --- | --- | --- | --- | --- |
| Control | -1.06±12.49 | -0.49±11.71 | 1.52±8.79 | 0.95±12.66 | 1.00±12.41 |
| 2 Weeks | -2.14±1.49 | -3.39±1.40 | -0.72±1.35 | -3.78±1.27 | -1.56±0.79 |
| 4 Weeks | -1.47±1.29 | -1.43±1.07 | -0.22±0.45 | -2.50±1.55 | -0.73±0.59 |

***Table S4:*** ***Mean and standard deviation of the intercept p_0,S_ of the lobule based linear model fitting for steatosis vs CYP/GS.*** *The distribution of p_0,s_ is less pronounced than for m_s_. There is a notable difference from normal to the other groups but little difference among those.*

| Mouse | GS *p_s_*_, 0_ | CYP1A2 *p_s_*_, 0_ | CYP3A4 *p_s_*_, 0_ | CYP2E1 *p_s_*_, 0_ | CYP2D6 *p_s_*_, 0_ |
| --- | --- | --- | --- | --- | --- |
| Control | 0.22±0.25 | 0.31±0.24 | 0.59±0.24 | 0.38±0.27 | 0.40±0.25 |
| 2 Weeks | 0.40±0.17 | 0.82±0.15 | 0.76±0.14 | 0.84±0.18 | 0.84±0.08 |
| 4 Weeks | 0.43±0.19 | 0.77±0.14 | 0.74±0.10 | 0.93±0.25 | 0.84±0.08 |
